# Ethical implications related to processing of personal data and artificial intelligence in humanitarian crises: a scoping review

**DOI:** 10.1186/s12910-025-01189-2

**Published:** 2025-04-15

**Authors:** Tino Kreutzer, James Orbinski, Lora Appel, Aijun An, Jerome Marston, Ella Boone, Patrick Vinck

**Affiliations:** 1 Kobo, Cambridge, MA 02139 USA; 2 The Montreal Children’s Hospital, McGill University Health Centre, Montreal, QC H4A 3J1 Canada; 3 The Dahdaleh Institute for Global Health Research, York University, Toronto, ON M3J 1P3 Canada; 4 Department of Family and Community Medicine, Temerty School of Medicine, University of Toronto, Toronto, ON M5S 1A8 Canada; 5 Munk School of Global Affairs and Public Policy, University of Toronto, Toronto, ON M5S 3K7 Canada; 6 Faculty of Health, York University, Toronto, ON M3J 1P3 Canada; 7 KITE, University Health Network, Toronto, ON M5G 2A2 Canada; 8 Michael Garron Hospital, Toronto, ON M4C 3E7 Canada; 9 Department of Electrical Engineering and Computer Science, York University, Toronto, ON M3J 1P3 Canada; 10 Harvard Medical School, Boston, MA 02115 USA; 11 Harvard TH Chan School of Public Health, Boston, MA 02115 USA; 12 Department of Emergency Medicine, Brigham and Women’s Hospital, Boston, MA 02115 USA; 13 Harvard Humanitarian Initiative, Cambridge, MA 02138 USA

**Keywords:** Humanitarian data, Ethical issues, Artificial intelligence (AI), Data processing, Ethical risks, Scoping review, Bioethical principles, Resource distribution

## Abstract

**Background:**

Humanitarian organizations are rapidly expanding their use of data in the pursuit of operational gains in effectiveness and efficiency. Ethical risks, particularly from artificial intelligence (AI) data processing, are increasingly recognized yet inadequately addressed by current humanitarian data protection guidelines. This study reports on a scoping review that maps the range of ethical issues that have been raised in the academic literature regarding data processing of people affected by humanitarian crises.

**Methods:**

We systematically searched databases to identify peer-reviewed studies published since 2010. Data and findings were standardized, grouping ethical issues into the value categories of autonomy, beneficence, non-maleficence, and justice. The study protocol followed Arksey and O’Malley’s approach and PRISMA reporting guidelines.

**Results:**

We identified 16,200 unique records and retained 218 relevant studies. Nearly one in three (*n* = 66) discussed technologies related to AI. Seventeen studies included an author from a lower-middle income country while four included an author from a low-income country. We identified 22 ethical issues which were then grouped along the four ethical value categories of autonomy, beneficence, non-maleficence, and justice. Slightly over half of included studies (*n* = 113) identified ethical issues based on real-world examples. The most-cited ethical issue (*n* = 134) was a concern for privacy in cases where personal or sensitive data might be inadvertently shared with third parties. Aside from AI, the technologies most frequently discussed in these studies included social media, crowdsourcing, and mapping tools.

**Conclusions:**

Studies highlight significant concerns that data processing in humanitarian contexts can cause additional harm, may not provide direct benefits, may limit affected populations’ autonomy, and can lead to the unfair distribution of scarce resources. The increase in AI tool deployment for humanitarian assistance amplifies these concerns. Urgent development of specific, comprehensive guidelines, training, and auditing methods is required to address these ethical challenges. Moreover, empirical research from low and middle-income countries, disproportionally affected by humanitarian crises, is vital to ensure inclusive and diverse perspectives. This research should focus on the ethical implications of both emerging AI systems, as well as established humanitarian data management practices.

**Trial registration:**

Not applicable.

**Supplementary Information:**

The online version contains supplementary material available at 10.1186/s12910-025-01189-2.

## Background

Humanitarian organizations work in challenging settings and with limited funding to provide life-saving aid. However, resources for providing this assistance are far from sufficient. By the end of 2024, donor governments provided US $21.2 billion to help 198 million people in 72 countries—a significantly smaller amount than the US $49.6 billion required to assist all 323 million people in need of humanitarian assistance for that year [1]. This considerable shortfall highlights the urgent need to better assess humanitarian needs and to do so at minimal cost.

The aim of this review is to map the range of ethical issues that have been raised in the academic literature regarding data processing of people affected by humanitarian crises. Humanitarian organizations rely on processing increasingly large amounts of data to inform their operations, much of which is collected directly from affected populations (e.g., through registrations, household surveys, or cash disbursements). At the same time, the people working for these organizations have themselves often become targets of kidnappings and killings, leading organizations to increasingly resort to remote methods of managing operations and collecting data from affected people [2, 3]. The COVID-19 pandemic has accelerated the trend of increased use of remote methods [4, 5]. This combination of factors has led to an exponential increase in the amount of personal data that is being distributed, stored, and analyzed in various locations around the world. At the same time, humanitarian organizations are continuously seeking innovations involving information and communication technologies (ICT) in the pursuit of operational gains in effectiveness and efficiency. This practice is expected to accelerate with the increasing sophistication of artificial intelligence (AI) technologies in the health and humanitarian sectors.

### Definitions

A review by Schofield et al. [6] found that the vast majority of included studies discussing “ethical challenges” in healthcare had failed to include an explicit definition of how that term was understood by the respective authors, leading to potential misunderstandings and ambiguity. This section, therefore, will first provide working definitions for the key terms and concepts discussed in this study. Humanitarian assistance is understood here to refer to coordinated actions that save lives and alleviate suffering of crisis-affected populations [7]. It also includes “protection”, which “encompasses all activities aimed at obtaining full respect for the rights of the individual in accordance with the letter and the spirit of the relevant bodies of law” [8]. Humanitarian crises are defined here as a “series of events representing a critical threat to the health, safety, security or wellbeing of a community, usually over a wide area” [9]. For the purposes of this study, data processing is understood as: “Any operation or set of operations which is performed on data or on sets of data, whether or not by automated means, such as collecting, registering, storing, adapting or altering, cleaning, filing, retrieving, using, disseminating, transferring and retaining or destroying” [10]. 

Drawing on Slim’s analysis [11], ethical issues in humanitarian contexts are defined as the dilemmas that arise when humanitarian workers’ values—such as compassion, fairness, and the duty to alleviate suffering—come into conflict with the complex realities of operating in conflict zones and natural hazard settings, necessitating ongoing negotiation between moral aspirations and practical constraints. This also reflects Beauchamp and Childress’ observation that “moral problems arise when obligations, rights, goods, or ideals conflict and require resolution through moral reasoning” [12].

### Context

Humanitarian organizations turning to new or existing digital tools to collect, store, or analyze data more efficiently may knowingly or inadvertently introduce new ethical issues affecting people who are already vulnerable [13]. In particular, several new technologies and tools have provoked deeper ethical discussions [14, 15], including biometrics [16, 17], location data [18], and “big data” [19], as well as drones [20–22] and social media and crowdsourcing platforms [23, 24]. Scholars and humanitarians are increasingly highlighting concerns in specific circumstances such as refugee registration [25–27], health emergency response [28, 29], and data sharing with governments, private corporations, and other third parties [30]. In practice, ethical decisions are made—knowingly or unknowingly—on a daily basis about what data to collect, which tools to use, or how and with whom to share this information to avoid adverse consequences [31, 32]. Organizations rarely choose to forego new tools altogether, such as Oxfam’s decision in 2015 to halt the use of biometrics in its programs in order to assess the potential risks [33]. Rather, some organizations are more likely to invest in new innovations without considering, weighing, or fully grasping the long-term ethical issues [34].

However, in light of these challenges, more guidelines are now being produced for the ethical processing of data for humanitarian assistance purposes, with the goal of minimizing or eliminating risks to vulnerable people. Notable examples include *Data Responsibility in Humanitarian Action* by the Inter-Agency Standing Committee (IASC) [35], which provides a comprehensive operational framework as well as practical assessment tools. In recent years, the Centre for Humanitarian Data has developed several relevant resources, including *Data Responsibility Guidelines* which provide principles and tools for managing data related to the vulnerabilities and needs of people in humanitarian situations, as well as data about operational contexts and response activities [10]. Several humanitarian organizations have created their own internal guidance on this subject, such as the *Handbook on Data Protection in Humanitarian Action* by the International Committee of the Red Cross (ICRC) [36]. Similarly, regulatory environments are changing in many countries (such as the European Union’s General Data Protection Regulation, GDPR), which have moved many humanitarian organizations to change their approaches to data processing in order to improve data privacy [37]. Focusing on the issue of ethical design of new tools, Krishnaraj et al. [38] have created practical guidelines that aim to mitigate risks as early as possible [39]. But the speed of technological innovation means that such guidance can quickly become out of date as new data technology tools appear and organizations respond to new circumstances. For instance, the rapid development of track-and-trace apps and other digital surveillance tools during the COVID-19 pandemic forced humanitarian organizations to adapt existing frameworks to address emergent privacy concerns [40].

AI systems that use machine learning and other methods for automating data processing are ushering in a completely new set of ethical issues that humanitarian organizations will have to confront [41]. Innovations using AI in the medical and health sectors have been growing significantly for years and are showing promise, such as in the discovery of new classes of antibiotics [42]. At the same time, large language models such as ChatGPT [43] that excel at generating and summarizing human language are introducing novel ethical issues [44], including in the health and medical sectors [45, 46].

Although a considerable number of studies discuss the ethics of using various technologies in humanitarian assistance, to date, no comprehensive review of relevant ethical issues has been published.

### Humanitarian and technical nomenclatures

Conducting this type of review is challenging due to the wide-ranging nature of humanitarian assistance, lack of well-defined nomenclature for data processing technologies and activities, and because relevant research may be published in the intersecting fields of ethics research, design, engineering, health, medicine, geography, development, social science, and technology research, among others. Previous scoping reviews focusing on humanitarian assistance only addressed more limited contexts or topics, such as disasters [47], displaced populations [48–51], cash transfers [52, 53], the use of drones [54, 55], or the phasing down and closing of humanitarian projects [56]. Other relevant studies have included scoping reviews of digital health tools and interventions in conflict settings and public health emergencies [57–59], a literature review focused on social media and privacy issues (based on literature published between 2013 and 2014 [60]), and systematic reviews covering digital innovation in humanitarian assistance, including deep learning and data-driven decision-making [61, 62]. However, we have not found a sufficiently comprehensive set of keywords that could be used to search databases for any of this study’s three inclusion criteria (people affected by humanitarian crises, processing data for humanitarian assistance, and meaningful discussion of ethical issues). In response, we developed a more inclusive set of search terms, drawing from the broad field of humanitarian assistance and intersecting academic disciplines, to capture the diverse range of relevant studies.

### Ethical frameworks

Another distinct challenge is the lack of established ethical categories or theories used by studies discussing ethics in the humanitarian sector [54]. Slim shows how modern humanitarian practice draws on an array of ethical frameworks, principles, and guidelines that were developed over the past decades by humanitarian practitioners [11]. Formally adopted by the International Red Cross and Red Crescent Movement in 1965 as part of a larger framework [63, 64], the four humanitarian principles (humanity, impartiality, independence, and neutrality) are now widely used among many humanitarian organizations [see, for example, 65], in international law [66], as well as in ethical codes attempting to guide the actions of the humanitarian sector as a whole [see, e.g., 67, 68]. Since then, these principles have been reexamined and reinterpreted as crises evolve, including in the 2015 thematic issue of the International Review of the Red Cross [69]. However, previous studies have shown the difficulty of applying these humanitarian principles in everyday practice [70], in guiding the use of information technology [71], or in mapping humanitarian organizations’ ethical obligations [72]. In particular, the broad *humanity* “principle” has been argued as being better understood as an absolute moral value rather than an ethical principle [10; see also, 73].

Other sectors have developed approaches in parallel to humanitarian response. While ethical principles such as justice and beneficence have been discussed since Ancient Greek philosophy and in other traditions, the post-World War II period saw a focused effort to systematically define ethical principles for medical practice and research, responding to growing awareness of the need to protect patient rights and dignity in modern medical practice. Among others, this included the Nuremberg Code [74], the 1964 Declaration of Helsinki [75], and the Belmont Report [76]. In this context, Beauchamp and Childress [12] developed their influential framework of four ethical value categories: *autonomy*, *beneficence*, *non-maleficence*, and *justice*, that continue to guide clinical decision-making and research practices.

Due to the challenges in applying humanitarian principles, many studies use these four ethical value categories as a practical framework for addressing ethical issues in humanitarian practice [77, 78]. We chose to use these four ethical value categories, defined in Table 1, to organize the ethical issues identified in the literature, linking the emerging field of humanitarian ethics with the established advances in bioethics and research ethics.

**Table 1:** Definitions of each ethical value category, based on Beauchamp and Childress [12]

Principle	Definition
Respect for autonomy	Respecting the decision-making capacities of autonomous persons
Beneficence	Providing benefits and balancing benefits against risks and costs
Non-maleficence	Avoiding the causation of harm
Justice	Distributing benefits, risks, and costs fairly

The aim of this review is to map the range of ethical issues that have been raised in the academic literature regarding data processing relevant to people affected by humanitarian crises. This study contributes to the existing academic discussion in three important ways. First, it presents the first comprehensive review of the ethical considerations in processing data from individuals affected by humanitarian crises, addressing a significant gap in the literature. Second, this review addresses the challenges of fragmented terminology by establishing an evidence-based search strategy to cover topics at the intersection of humanitarian assistance, data processing, and ethical implications. Third, this study introduces a clear, transparent framework for defining what constitutes a “humanitarian crisis,” providing a consistent basis for including or excluding studies focusing on disaster events, categorized by a country’s income level, which may help avoid subjective biases in research selection for future studies.

## Methods

### Study protocol

We chose to conduct a scoping review as this method is best suited for generating a broad overview of relevant evidence, examining emerging areas of research, clarifying key concepts, and identifying gaps in the literature [79]. A study protocol was developed prior to data collection and screening, following the scoping review method established by Arksey and O’Malley [80], further refined by Levac et al. [81], and aligned with the framework maintained by the Joanna Briggs Institute [82]. The protocol was revised based on feedback received from the research team and incorporated the results from a pilot conducted for this study. It follows the Preferred Reporting Items for Systematic reviews and Meta-Analyses extension for Scoping Reviews (PRISMA-ScR) reporting guidelines [83]. The final version of the PRISMA-ScR checklist and the study protocol are available in Supplementary Material 1 and 2, respectively.

### Identifying the research question

The specific research questions of this scoping review were:

1. Which ethical issues have been raised in the literature related to processing data from people affected by humanitarian crises in order to inform humanitarian assistance?
2. To what extent do real-world examples of ethical issues reflect the concerns presented in the literature?
3. Which technologies were the focus of concern over these ethical issues?

### Eligibility criteria

The following eligibility criteria for the selection of relevant studies were established a priori as per the categories and requirements for scoping review protocols [83].

#### Condition/Domain

Ethical issues stemming from the processing of data relating to people affected by a humanitarian crisis with the explicit goal or potential of informing humanitarian assistance.

#### Population

People affected by a humanitarian crisis, including refugees and transborder migrants fleeing from such a crisis—regardless of their current location. We also included studies that concern humanitarian assistance (including related fields such as disaster response or emergency management) that are global in scope.

Studies about disasters were only included if the study focused on events in low- or lower middle-income countries, defined as countries classified as such by the World Bank at least once between 2011 and 2024 [84]. This approach was used to distinguish responses in humanitarian contexts from responses to natural hazards in higher-income countries, which are typically considered more resilient and less likely to escalate to a humanitarian crisis [see, e.g., 85]. The Ebola outbreak in West Africa (2014-2016) was included as it was widely considered to be a humanitarian crisis in scope [86]. We used the Financial Tracking Service by the United Nations Office for the Coordination of Humanitarian Affairs (OCHA) [87] to judge if an event should be considered a humanitarian crisis (defined as whether a given country was a recipient of humanitarian aid in the same year). Studies primarily focusing on COVID-19 responses were included only if the country or territory in question was already considered to be in a humanitarian crisis based on our criteria.

#### Interventions

Data processing relating to people affected by a humanitarian crisis with the explicit goal or potential of informing humanitarian assistance. Excluded were studies that focus on technologies that do not process data on affected people, such as robotics for clearing debris or land mines, algorithmic models for predicting the occurrence or impacts of natural hazards, or tools used for planning humanitarian logistics (e.g., relief networks, supply chains, and resource scheduling).

#### Outcomes

Studies that investigate ethical issues stemming from the processing of data (as defined above) were included only if they contained a significant discussion about this subject. During the screening stage, studies were eligible for inclusion if the abstract referenced or mentioned potential ethical issues. During the full text review, this was assessed qualitatively by two reviewers.

#### Study designs

All study designs were eligible for inclusion. Non-peer reviewed studies were excluded to ensure a robust foundation for formulating evidence-based recommendations, maintain feasibility given the challenges with differing nomenclature and the absence of a unified grey literature database, support replicability through well-documented academic search strategies, and align with the study’s focus on ethical concerns in academic literature related to humanitarian data processing.

#### Context

For feasibility reasons, we restricted the review to studies published after 1 January 2010.

#### Setting

Studies in all countries or territories affected by a humanitarian crisis (or relevant host countries for refugee or cross-border migrant or displaced populations) were included, as defined above.

### Search strategy and information sources

Comprehensive literature searches of electronic databases were conducted on 31 March 2020 (for studies published between 2010 and 2019) and 2 September 2024 (for studies published between 2020 and August 2024), using Ovid, Ebsco, Web of Science, and Proquest to search 20 databases for relevant studies. Only studies published in English, French, or Spanish were included.

As recommended by the scoping review guidelines described above, keywords were selected and piloted in multiple iterations to identify all relevant articles. We had previously identified 34 studies, and these were used as a minimum search target. After an initial search showed that only 13 were included, we repeated the database search over several iterations with additional terms until all 34 studies were reflected in the results. This yielded additional keywords such as “risks” and “challenges” to represent ethical challenges, as well as “innovation” and “experimentation” which are sometimes used to refer to data processing activities. Further, careful searching for terms such as “acute malnutrition” or “forcibly displaced population” was also found to describe specific phenomena in a humanitarian crisis without using terms such as “refugees” or “humanitarian” in the study’s metadata. Likewise, to find all studies that discuss processing data of affected people, we iteratively expanded our search terms to include specific technologies (e.g., biometrics, remote sensing), emerging practices (e.g., remote management, crowdsourcing), or shorthand keywords introduced by researchers (e.g., experimentation, crisis informatics, innovation). Finally, to include studies related to humanitarian assistance during natural hazards, we included disaster-related keywords such as “disaster relief,” “disaster response,” and “disaster assistance,” among other combinations. A sample of the search strategy for the Ovid databases is displayed in Table 2. The complete search syntax for each database can be found in Supplementary Material 3.

**Table 2:** Search strategy for Ovid databases

Concept		Keyword and syntax
Humanitarian assistance	1	humanitarian*.tw.
	2	relief work.tw.
	3	aid work.tw.
	4	(disaster? Adj (relief or response? Or assistance)).tw.
	5	emergency relief.tw.
	6	((conflict? Or war?) adj10 (human rights or public health)).tw.
	7	(ebola adj6 (west africa or sierra leone or liberia or guinea or 2014 or 2013)).tw.
	8	acute malnutrition.tw.
	9	(refugee* adj2 (camp* or assistance or population?)).tw.
	10	(displace* adj2 (forced or forcibly or population? Or human? Or internal*)).tw.
	11	(((population? Or person* or communit*) adj3 affected) adj1 (conflict? Or violence)).tw.
	12	or/ 1–11
	13	(cris? s or emergenc* or disaster? or “natural hazard?“).tw.
	14	humanitarian*.af.
	15	13 and 14
	16	12 or 15
ICT for data collection	17	ict.tw.
	18	technolog*.tw.
	19	((data or information) adj2 (system* or manage* or collection or analys? s or process*)).tw.
	20	(blockchain or distributed ledger).tw.
	21	(a.i. or artificial intelligence or machine learning or algorithm*).tw.
	22	biometric*.tw.
	23	smartphone app*.tw.
	24	remote sensing.tw.
	25	analytics.tw.
	26	digital*.tw.
	27	experimentation.tw.
	28	automat*.tw.
	29	innovation?.tw.
	30	remote management.tw.
	31	cyber.tw.
	32	big data.tw.
	33	(sms or text messag* or interactive voice recognition or online survey*).tw.
	34	(kobotoolbox or kobo or odk or open data kit).tw.
	35	crowdsource*.tw.
	36	social media.tw.
	37	crisis adj (informatics or data or map*).tw.
	38	digiti? ation.tw.
	39	datafication.tw.
	40	or/ 17–39
Ethical concerns	41	concern?.tw.
	42	risk?.tw.
	43	challenge?.tw.
	44	harm?.tw.
	45	privacy.tw.
	46	protection?.tw.
	47	humanitarian adj (principle? Or standard? Or guideline?).tw.
	48	problem?.tw.
	49	bias?.tw.
	50	ethic*.tw.
	51	consequence?.tw.
	52	critique?.tw.
	53	insecurity.tw.
	54	implications.tw.
	55	peril?.tw.
	56	impact?.tw.
	57	or/ 41–56
		16 and 40 and 57

### Study selection

Study selection and coding were done using the DistillerSR systematic review software [88]. Using the a priori eligibility criteria, we developed questionnaires for selecting citations during discrete title, abstract, and full text review stages. Two reviewers independently selected studies during each screening stage.

Regular meetings were held to discuss rating discrepancies and to compare working definitions during the review of the first 1,000 references in the title screening stage and for the first 100 references during the abstract screening stage. Any conflicts during the title and abstract screening stages were included in the full text review. In the full text screening stage, daily meetings were held during the review of the first 20 references to discuss rating discrepancies and to improve working definitions of terms. Rating discrepancies were resolved by discussion, and in five cases, by using a third adjudicator.

### Data collection process

For included studies, we extracted details on study characteristics (year of publication, countries of all authors, author organization types), population characteristics (type of humanitarian crisis), intervention characteristics (purpose of data processing, technologies described), and outcomes (specific ethical issues identified, whether studies used real-world examples to identify issues). We coded author organization types for all listed affiliations but extracted author country only from the first-listed affiliation. For each country, we additionally tabulated the geographic region and income level, using the 2024 World Bank classification scheme [84].

The data extraction form was created in the DistillerSR software. It was then piloted based on a random sample of 10 included studies and modified based on discussions and feedback from the two reviewers. As per the study protocol, since the number of included citations was greater than 30, data extraction was done by one reviewer and verified by another. The data extraction form included several pre-coded ethical issues, but additional emergent issues could be entered qualitatively in text format.

### Synthesis

We summarized results quantitatively (using frequencies) and qualitatively (using descriptive analytics). We analyzed and coded the ethical issues related to data processing that were entered in text form using SPSS 25. Specific issues described by authors could be assigned to one or more categories of ethical issues. Issue codes were updated iteratively and recursively by creating new codes based on new observations and through constant retrospective reviews of previously collected data. In some cases, rarely-mentioned codes were also merged retrospectively to limit the size of the final list of issues. The ethical issues mentioned in each study were then grouped into the ethical value categories of autonomy, beneficence, non-maleficence, and justice based on the category deemed most affected.

## Results

### Literature search

**Fig. 1:**
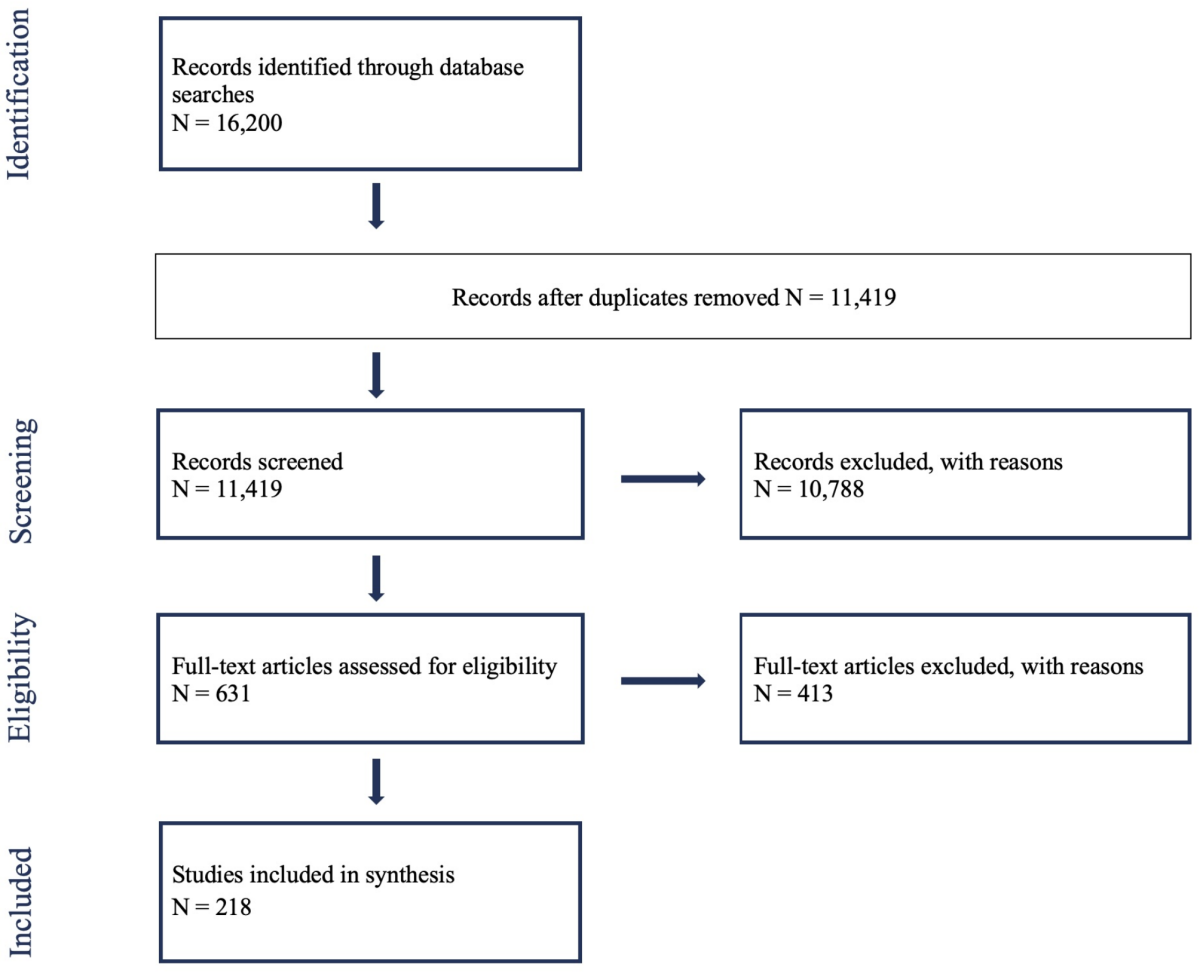
PRISMA diagram

The database literature search returned 16,200 citations (see Fig. 1). After removing duplicates, 11,419 were included for screening. 10,788 were excluded during the screening stage. After reviewing full texts of 631 potentially relevant studies, 413 were excluded. As a result, 218 were included in this scoping review (full list of citations listed in Supplementary Material 4).

### Study characteristics

**Fig. 2:**
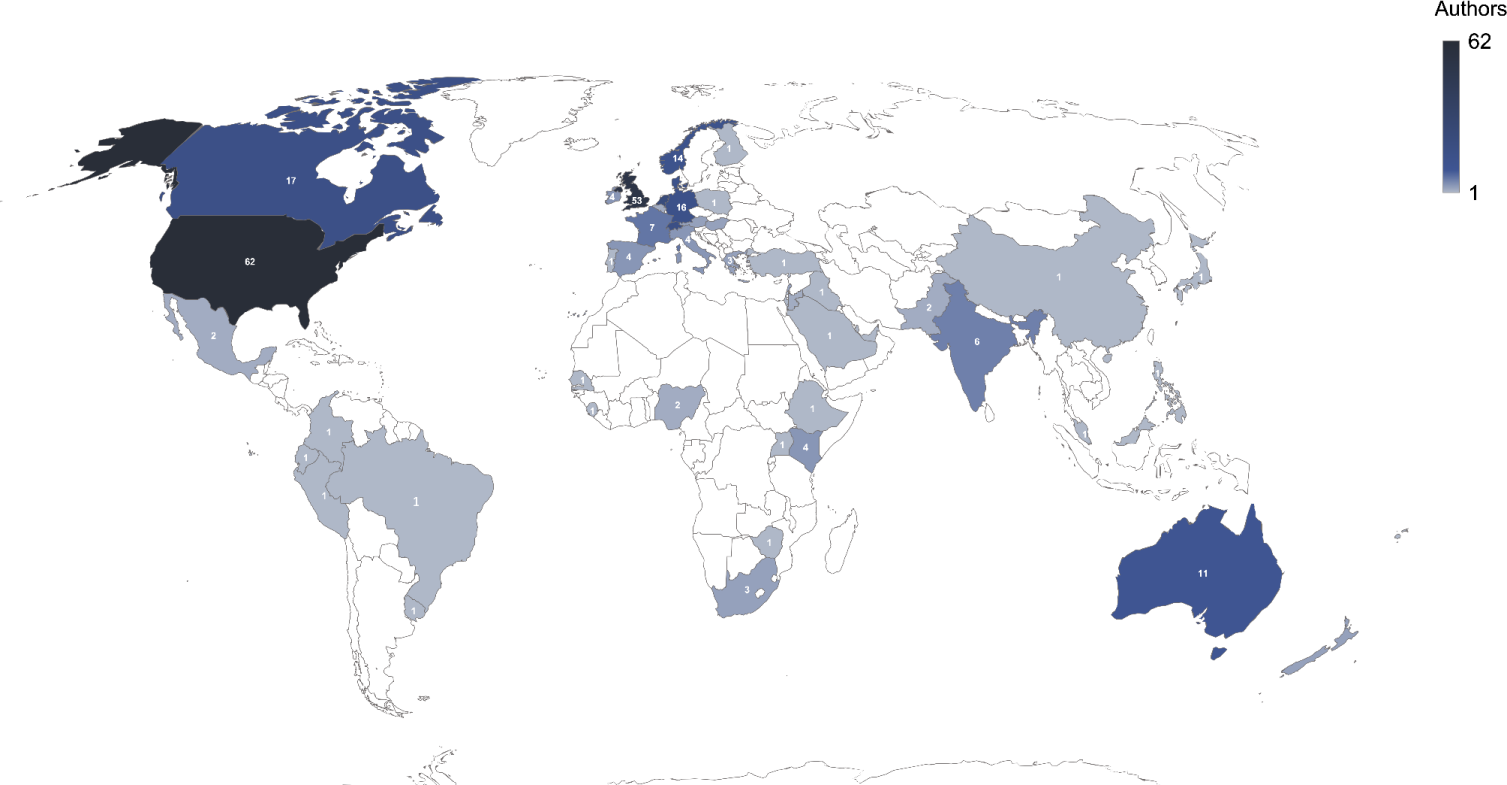
Map showing the number of studies per country based on authors’ affiliation

The included 218 studies were published between January 2010 and August 2024, as shown in Table 3. The majority (*n* = 191) were published after 2015, and the most common publication year was 2023 (*n* = 34). Most were written by authors based in Europe and Central Asia (*n* = 137) and North America (*n* = 75), while only a small number of studies included authors from East Asia and the Pacific (*n* = 19), South Asia (*n* = 7), Sub-Saharan Africa (*n* = 13), Middle East and North Africa (*n* = 10), and Latin America and the Caribbean (*n* = 4), as shown in Fig. 2, which displays the number of studies by country. Sixty-two studies included an author from the United States while about one quarter (*n* = 53) included an author from the United Kingdom (as studies typically have multiple authors, they may be included in the counts for more than one country). Overall, 206 studies included at least one author from a high-income country, compared to smaller numbers from upper middle-income countries (*n* = 16), lower middle-income countries (*n* = 17), or low-income countries (*n* = 4). The vast majority (*n* = 207) of studies included at least one author from an academic institution, while only seven studies included at least one author affiliated with a humanitarian organization.

**Table 3:** Study characteristics (*n* = 218)

Characteristic	Count (%)
*Year of publication*	
2010	2 (1%)
2012	1 (0%)
2013	5 (2%)
2014	9 (4%)
2015	10 (5%)
2016	20 (9%)
2017	10 (5%)
2018	13 (6%)
2019	29 (13%)
2020	20 (9%)
2021	20 (9%)
2022	21 (10%)
2023	34 (16%)
2024	24 (11%)
*Region represented by authors*	
Europe & Central Asia	137 (63%)
North America	75 (34%)
East Asia & Pacific	19 (9%)
South Asia	7 (3%)
Sub-Saharan Africa	13 (6%)
Middle East & North Africa	10 (5%)
Latin America & Caribbean	4 (2%)
*Country income level based on author location*	
High income	206 (94%)
Upper middle-income	16 (7%)
Lower middle-income	17 (8%)
Low-income	4 (2%)

### Type of humanitarian crisis

Similar numbers of studies focused on or included examples of disasters and armed conflict (*n* = 61 and *n* = 66, respectively), with many studies focusing on more than one setting, as shown in Table 4. Of the 218 studies selected, 75 discussed people displaced by a humanitarian crisis. Meanwhile, 60 were general in nature and only discussed the fields of humanitarian assistance, emergency management, or disaster response without providing specific examples.

**Table 4:** Types of humanitarian crises discussed (*n* = 218)

Type of humanitarian crisis	Count (%)
Refugees or migrants who fled a humanitarian crisis	75 (34%)
Armed conflict	66 (30%)
Disaster	61 (28%)
Not specified	60 (28%)

### Purpose of data processing

While most studies reported more than one purpose, the most common was conducting assessments (*n* = 53), such as needs assessments or damage surveys (see Table 5). Fifty studies examined different forms of case management (e.g., refugee registrations), while 40 considered the delivery of assistance and 31 discussed handling of medical or public health data. Forty-seven did not specify any reasons for data processing but instead discussed in theoretical terms the use of information and communication technologies or data processing in humanitarian assistance.

**Table 5:** Data processing purposes and technologies described by studies (*n* = 218)

Purposes of data processing	Count (%)
Assessment (of needs, damage, etc.)	53 (24%)
Registration / case management	50 (23%)
Delivery of assistance	40 (18%)
Medical care or public health	31 (14%)
Forecasting / modeling / early warning	28 (13%)
Human rights violations	20 (9%)
Cash transfer	19 (9%)
Other	17 (8%)
Accountability (complaints, feedback collection, etc.)	15 (7%)
Logistics	12 (6%)
Search and rescue	11 (5%)
Not specified	47 (22%)

### Technologies described

The most commonly described technologies used for data processing were social media (discussed by 74 studies) and various tools and technologies related to AI, including the use of algorithms and machine learning (*n* = 66). “Big data” (*n* = 56), mapping and other forms of geographic information systems (GIS; *n* = 52), and crowdsourcing (*n* = 48) were also routinely included.

Nearly one-fifth (*n* = 42) discussed the collection of biometrics (typically, fingerprint or iris scans), while fewer studies described the use of unmanned aerial vehicles (UAV) for the collection of humanitarian data (*n* = 32) or the capture of satellite images for humanitarian assistance (*n* = 31). Other technologies cited are shown in Table 6 which shows the distribution of technologies described in the studies, with many studies examining multiple technologies simultaneously. Seventy-four studies did not discuss any specific technologies used for data processing.

**Table 6:** Technologies described by studies (*n* = 218)

Specific technologies described	Count (%)
Social media	74 (34%)
AI / algorithms / machine learning	66 (30%)
Big data	56 (26%)
Mapping / GIS	52 (24%)
Crowdsourcing	48 (22%)
SMS or private messaging software	47 (22%)
Information systems	45 (21%)
Biometrics	42 (19%)
UAV	32 (15%)
Satellite imagery	31 (14%)
Cash distribution	25 (11%)
Medical data	21 (10%)
Blockchain / distributed ledger technology	17 (8%)
Data storage	14 (6%)
Call data records	11 (5%)
Computer-assisted personal interviewing	7 (3%)
Not specified	74 (34%)

### Ethical issues identified

As shown in Table 7, we identified 22 ethical issues in the studies under investigation, which were grouped according to the four previously identified bioethical values categories. Seven issues were attributed to the ethical value category of autonomy, five to beneficence, six to non-maleficence, and four to justice. On average, studies cited around seven different ethical issues each (*M* = 6.7, *SD* = 3.08), ranging from approximately one for justice issues (*M* = 1.3, *SD* = 0.97) to more than two for non-maleficence issues (*M* = 2.22, *SD* = 1.27; see Table 8). The vast majority of studies mentioned issues related to non-maleficence (*n* = 199) and beneficence (*n* = 191). Slightly fewer studies discussed issues concerning justice (*n* = 174) and autonomy (*n* = 146).

**Table 7:** Ethical issues identified (*n* = 218)

Ethical issues identified		Count (%)
Autonomy	Lack of consent: Data is collected without informed consent	91 (42%)
	Data agency: People do not have the right to control, access, or delete their data	50 (23%)
	Participation: People/communities are not involved in decisions to use of new/experimental technologies for collecting data	50 (23%)
	Undisclosed use: Data may be used beyond purposes for which they were collected	40 (18%)
	Lack of respect: People/communities are not treated with respect	37 (17%)
	Autonomy: Unwillingness to share data does not lead to disadvantages (e.g., exclusion from assistance or protection)	35 (16%)
	Lack of group agency: Processed information is not available to affected communities	8 (4%)
	*Any implication related to Autonomy*	146 (67%)
Beneficence	Unreliability: Processed data is inaccurate and does not sufficiently reflect reality to inform assistance	108 (50%)
	Dependence: Data is processed with the assistance of a political, economic, or military entity	107 (49%)
	Non-neutrality: Data is processed in a way that benefits or appears to benefit one side of the conflict over the other	67 (31%)
	Ineffective or inefficient: Not producing expected result, unmet expectations	56 (26%)
	Lack of action: Processed data is not utilized to inform assistance to the affected person/community	42 (19%)
	*Any implication related to Beneficence*	191 (88%)
Non-maleficence	Privacy: Personal/sensitive data is shared with third parties	134 (61%)
	Harm: People suffer physical or psychological harm as a result of data processing	106 (49%)
	Data security: Personal/sensitive data is not protected against malicious actors	103 (47%)
	Power imbalance: Data processing reinforces or worsens a lack of power of affected people	93 (43%)
	Excess: More data was collected than necessary	28 (13%)
	Redress/rectification: People do not have the ability to correct wrong information about them or receive compensation	16 (7%)
	*Any implication related to Non-maleficence*	199 (91%)
Justice	Bias: Data is processed in a way that may (dis)advantage some people disproportionate to their humanitarian needs	122 (56%)
	Unequal access to technology / exclusion from data collection	75 (34%)
	Lack of accountability: Endangering (or not protecting) rights; absolving responsibility	52 (24%)
	Unfair distribution of risks and benefits	37 (17%)
	*Any implication related to Justice*	174 (80%)

**Table 8:** Number of ethical issues cited by ethical value category (*n* = 218)

Bioethical value category	mean, SD (min to max)
*All ethical value categories*	*6.7*,* 3.08 (1 to 16)*
Autonomy	*1.44*,* 1.38 (0 to 7)*
Beneficence	*1.75*,* 1.12 (0 to 5)*
Non-maleficence	*2.22*,* 1.27 (0 to 6)*
Justice	*1.3*,* 0.97 (0 to 4)*

The most frequently cited ethical issue categorized under the value category of autonomy was data being collected without sufficient informed consent (*n* = 91). For example, Shoemaker et al. [89] found through interviews with refugees that they are frequently being asked by humanitarian organizations to provide personal information that the respondents considered intrusive, without being offered a justification on why this was relevant.

Within the value category of beneficence, the ethical issue most frequently mentioned by studies was processed data being inaccurate and not sufficiently reflecting reality to inform assistance (*n* = 108). This is illustrated by Paul and Sosale [90], who highlight the challenges of using social media as a basis to inform humanitarian assistance. In an example the authors cite, the same information was re-posted multiple times by well-meaning users, making it difficult for emergency responders after a severe flooding event to identify new information that might require a team to be dispatched. Likewise, Tran et al. [91] show that misinformation posted to social media has significant negative impacts for humanitarian response and recovery.

Falling under the value category of non-maleficence, the most-cited ethical issue (*n* = 134) was privacy concerns in cases where personal or sensitive data may be shared with third parties. For example, Hayes and Kelly [92] discuss how personal requests for help that are aggregated by a crowdsourcing platform such as Ushahidi can make personal information publicly available, including to bad actors trying to exploit vulnerable people.

The most frequently mentioned ethical issue categorized under the justice value category was biased data processing leading to the inequitable distribution of humanitarian assistance compared to people’s needs (*n* = 122). For instance, Beduschi [93] considers how facial recognition software more frequently misidentifies women with darker skin and disabled persons using assistive equipment, meaning certain groups seeking aid to which they are entitled may have it denied based on their physical characteristics. The issue of biased data processing has become more pressing as more organizations turn to “big data” solutions for informing humanitarian assistance without properly understanding their limitations [94].

### Information sources for ethical issues

Slightly over half of studies (*n* = 113) cited at least one real-world example of an ethical issue, usually based on anecdotal information found in news reports or other published literature [see, e.g., 95, 96], with many studies providing more than one source for the ethical issues discussed (see Table 9). Over 85% of studies (*n* = 187) included ethical issues that were raised by interviews or other kinds of consultations with experts. Examples here include Shoemaker et al. [89], who conducted qualitative interviews with 198 refugees in Lebanon, Jordan, and Uganda, Vannini et al. [97], who interviewed nine representatives from organizations assisting transborder migrants in the United States, and Açιkyιldιz [98], who interviewed 17 humanitarian aid workers about their organizations’ use of biometric data. Fifteen studies included a systematic review of the literature.

**Table 9:** Information sources of ethical issues (*n* = 218)

Sources of ethical issues	Count (%)
Ethical concerns raised by interviews / expert consultations	187 (86%)
Specific instances of ethical issue	113 (52%)
Systematic review of the literature	15 (7%)

### Key results for studies discussing AI

Of the 66 studies that discuss the use of AI, all were published since 2014, with about three-quarters (*n* = 49) published since 2019 (see Supplementary Material 5 for all figures pertaining to the AI-related studies). The most common type of humanitarian crisis discussed in the 66 studies was disasters (*n* = 23), followed by people fleeing a humanitarian crisis (*n* = 18) and armed conflict (*n* = 16). The most common purposes for data processing were related to assessments (*n* = 20), delivery of assistance (*n* = 15), and registration and case management (*n* = 15). Many of the 66 studies related to AI discussed this technology in relation to “big data” (*n* = 29), social media (*n* = 28), and biometrics (*n* = 15). The majority mentioned ethical implications related to biased data processing that could lead to inequitable distribution of humanitarian assistance (*n* = 48). Privacy and unreliable data were also common ethical concerns (*n* = 41, respectively).

## Discussion

### Range of ethical issues

The aim of this review was to map the range of ethical issues that have been raised in the academic literature regarding processing data from people affected by humanitarian crises. This review identified 22 such ethical issues. Issues related to the value category of non-maleficence were brought up by the vast majority of studies (*n* = 199), which dovetails with a strong trend in the recent literature focusing on the imperative of “do no harm” in humanitarian assistance [99–101]. Within this value category, the risk of increasing harm (whether physical or psychological) as a result of data processing was mentioned by a high number of studies (*n* = 106).

Privacy concerns were cited by over 60% of studies (*n* = 134)—more commonly than all other issues—reflecting an increased awareness of this issue over recent years across organizations and the media. This emphasis held among studies discussing AI as a data processing technology, with 41 out of 66 (62%) mentioning privacy. This points to a significant worry across the humanitarian sector about the many ways in which personal data from affected people is being processed in a manner that may endanger their right to privacy, which is enshrined in the 1948 Universal Declaration of Human Rights [102]. Studies discuss a wide gamut of how personal privacy can be violated, including accidental or intentional sharing with third parties beyond what the affected person had agreed to during personal interviews, or if at all. Even in cases where informed consent was given, interviewees in vulnerable situations—or who lack understanding of sophisticated data management, access, and processing—may not understand all the potential ways their personal information may be used, stored, and accessed. Collecting and processing personal data from social media, by UAV, or public records (often under the “big data” category) that lack explicit consent are particularly problematic. Although the protection of privacy can be understood as an essential right to safeguard human dignity [103], more studies and initiatives in humanitarian assistance need to resolve the apparent conflict between the duty to protect privacy and the urgent duty to assist and protect those in danger [92].

Many studies pointed out that organizations frequently collect much more data than they need (*n* = 28) or are able to act on (*n* = 42). We consider the former a potential harm, as any excessive information increases risks to individuals’ privacy and security. Collecting data that is not used was linked to the ethical value of beneficence, as this implies that all information collected should have a concrete purpose related to informing humanitarian assistance. But even for data that were used for the intended purpose, nearly half (*n* = 108) of studies discussed that it may be too unreliable or inaccurate to adequately inform assistance programming.

A dominant theme emerged regarding insufficient consent mechanisms, which strongly relates to the ethical value category of autonomy. About 40% of studies (*n* = 91) mentioned that informed consent was either not provided by the affected population or was given without a full picture of how data would be processed or used. Forty studies cited that data might be used for reasons other than the original purpose for which consent may have been obtained. Related to the ethical value category of autonomy, more than 15% of studies (*n* = 35) mentioned that a refusal to provide information could lead to being excluded from receiving assistance. This issue is illustrated by Shoemaker et al. [89] who documented how refugees felt that they lacked a choice on whether or not to provide personal information to the UN High Commissioner for Refugees (UNHCR) as their ability to access assistance depended on it. Detailed guidance has been created by the International Rescue Committee on how to obtain proper consent [104], whereas the ICRC has published the legal basis for situations when data processing is permissible—even when consent cannot be assumed or obtained [36]. However, more work is clearly needed to train humanitarian professionals in these practices, and to monitor for better compliance with best consent practices as well as other minimal ethical guidelines. Existing guidance also needs to be updated to ensure the protection of private, personal, and demographically identifiable information that extends to population groups rather than individuals [105].

Directly related to the value category of justice, more than half (*n* = 122) of studies were concerned about data being processed in a way that may result in aid being distributed unevenly compared to people’s actual needs. This finding directly mirrors the importance of the humanitarian impartiality value category which refers to providing assistance solely based on need and regardless of personal preferences or discriminatory factors [64].

A cross-cutting issue was the potential of data processing to exacerbate power imbalances (mentioned by 93 studies), often due to an exclusion from data collection, given the unequal access to certain technologies (*n* = 75). In many cases, data processing was found to diminish the perceived neutrality of humanitarian organizations (*n* = 67) as data could be processed in a way that might benefit one side of the conflict over the other. Concerningly, nearly half (*n* = 107) of the studies found that humanitarian data processing often depends on potentially biased external entities (such as commercial entities, militaries, or foreign governments). This could be increasingly problematic for humanitarian organizations for multiple operational and ethical reasons, but particularly in conflict environments where the perception of independence is widely considered to be an essential humanitarian value category.

Another theme identified across many studies was that data processing did not follow the principles of Accountability to Affected People [106, 107], which manifested in various ways across several of the four bioethical value categories. For the value category of autonomy, 50 studies remarked that affected communities were not involved in decisions of whether to use experimental technologies for data collection, whereas a smaller number (*n* = 8) commented that processed information was not being made available to communities to allow for better group agency. Related to the value category of non-maleficence, 16 papers discussed people’s inability to rectify inaccurate information about them or receive any form of compensation. Finally, related to the value category of justice, about one in four (*n* = 52) studies found that data processing lacked accountability in terms of humanitarian organizations’ obligation to protect rights—or even pointed to ways that they may be violating these rights themselves. The Signal Code [108], first published by the Harvard Humanitarian Initiative in 2017, considers data agency and redress/rectification as crucial rights and proposes specific actions to safeguard them in practice. We propose extending this list to always include affected communities in decisions about sharing collected data and their involvement in decisions over experimental technologies.

About half of studies (*n* = 113) cited ethical issues that were rooted in real-life experiences whereas over 85% (*n* = 187) contained issues based on qualitative interviews or expert consultations. This signals that ethical issues have moved from theoretical concerns to actual incidents. However, it also reflects the large and diverse array of ethical issues that are emerging in connection with data processing in humanitarian crises which may first manifest as theoretical concerns before being validated as potentially negative consequences that can and do occur in real life.

### Geographic disparities

Geographically, publications were disproportionately from authors in high-income countries, primarily in Europe and North America, demonstrating a high level of interest in countries that have been the traditional source of most humanitarian funding but also of most technological innovation. Conversely, the limited number of authors from lower-middle income countries and the even smaller number of authors from low-income countries highlights the lack of published perspectives from countries most affected by humanitarian crises. The inclusion of only one study with an author from China may reflect that the large body of disaster related studies from Chinese authors published in English primarily discuss the response to domestic rather than foreign crises, or that ethical issues explored in this review may be more explored in Chinese language publications. People living in affected countries make up the vast majority of humanitarian organizations’ staff, which could be a potential boon to a more diverse authorship on this subject. However, given the very small number of studies with authors from a humanitarian organization, more efforts need to be made by publishers to invite and support submissions from humanitarian professionals.

We found that studies containing ethical discussions are often skewed towards investigating disaster contexts. The number of studies discussing disasters (*n* = 61) was about the same as the number discussing armed conflict (*n* = 66), even though by the end of 2023, 90% of displacement was caused by conflict [109]. This disproportionate focus may be due to disasters generating a higher level of media attention, as well as interest among technology enthusiasts, volunteers, and private companies—a trend identified by several studies [110–113]. Likewise, empirical research in conflict settings is far more difficult given the inherent security risks, which in turn limits the development of theories and academic discourse that rely on data from the field.

### Technology focus

Our study shows that academic papers often follow trends in innovation rather than investigating the most widely used technologies. For example, studies discussing the ethics of using “big data,” crowdsourcing, and other remote data collection technologies have been growing significantly in recent years. This trend exploded further during the COVID-19 pandemic as humanitarian organizations adopted remote data collection technologies at an even higher pace. For instance, open-source platforms including Ushahidi and Sahana were used during the pandemic to map services, respond to needs for medicine, and coordinate volunteers, among other activities [4]. Studies investigating these emerging trends are important for highlighting emerging ethical challenges, such as unequal aid distribution and barriers to accessing digital services among the most marginalized [40, 114].

We found that studies most commonly discussed activities involving the initial collection of data from affected populations, including assessments, registrations, and health interventions. To some extent, this reflects that a large number of studies investigated the use of crowdsourcing and social media to gain an understanding of a particular humanitarian crisis (see below). It may also reflect the increasing emphasis that humanitarian organizations and their donors have placed in recent years on establishing an “evidence base” before rolling out assistance programs [115, 116]. More research is needed to investigate the link between the potential increase of ethical risk and the push for collecting more needs assessment data.

Studies discussing social media (*n* = 74), mapping (*n* = 52), and crowdsourcing (*n* = 48) dominated, often due to the perceived lack of quality ground-validated data in humanitarian assistance. There were many use cases of social media, but the most-discussed application was mining public Twitter/X posts for clues on potential population needs. We also found that many studies focus on the potential use of other “new” technologies, especially if they can be used remotely to assess needs (e.g., satellite imagery, unmanned aerial vehicles, call data records). Crowdsourcing, a method of obtaining information from the general public [117], was discussed by almost a quarter of the studies. Many studies traced their enthusiasm for—or criticism of—crowdsourcing to the creation of the Ushahidi platform (mentioned by 32 studies) in 2007. Similarly, the emergence of digital platform-based volunteer networks since the 2010 Haiti earthquake [118, 119] can partially explain the large number of studies referencing these tools. As Burns [23] points out, such “digital humanitarianism” can produce narratives of “victims” who can only be saved by crowdsourcing and other software platforms.

Surprisingly, only seven studies mentioned computer-assisted personal interviewing (CAPI) tools such as KoboToolbox which has been adopted by a broad range of international and national humanitarian agencies as the tool of choice for humanitarian assistance [120, 121]. Similarly, storing and sharing personal data in spreadsheets was barely mentioned by studies as a cause for ethical concern, despite being the main data processing mechanism of choice for many humanitarian organizations [122]. Such low-tech data processing means are addressed in recent guidelines, for example, by giving guidance on how to remove sensitive data before sharing Excel files with others [10]. However, more research is needed on current practices and ethical risks associated with these commonly used technologies.

The ethical issues associated with biometrics were discussed by a significant number of studies, particularly for the registration of refugees and other migrants by organizations such as UNHCR (see, e.g., [25]). Hayes [123], for one, warns that humanitarian organizations may inadvertently aid states to surveil migrants and curtail irregular migration by collecting biometric data in their effort to efficiently provide assistance. In 2015, ethical concerns led Oxfam, one of the largest international humanitarian organizations, to put a moratorium on its use of biometrics in order to assess potential risks [33]. In 2021, this in turn resulted in the creation of a policy intended to ensure that the technology is used ethically within Oxfam’s operations [124].

Finally, ethics related to AI and similar technologies were mentioned significantly more frequently in recent years, with 38% of the included studies published since 2022 discussing AI. This trend correlates with a decrease in the number of studies focusing on “big data” (which has declined since its peak in 2019) and crowdsourcing (peaking in 2016), reflecting a growing interest in AI both within and outside humanitarian response. Whereas earlier studies before 2020 referred often vaguely to machine learning or the potential role of “algorithms,” more recent research focuses on practical applications. Relevant examples include studies exploring the risks of deploying AI in conflict zones while relying on private companies [125], embedding ethical principles in predictive tools for migration management [126], and addressing fairness in machine learning models given the concentration of actors in the Global North [127]. The growing sophistication of natural language processing has led to several use cases for humanitarian response, such as developing the HumBERT model for text classification and bias mitigation in crisis contexts [128], analyzing data from sources such as social media and Humanitarian Needs Overviews to monitor and anticipate crises [129], and enhancing needs assessments through large-scale analysis of interview responses [130]. Additionally, efforts to develop trusted human-AI networks highlight the need for robust ethical frameworks that prioritize collaboration and transparency in decision-making [131]. More theoretical and empirical research is needed to address gaps in understanding how these rapidly evolving technologies can be safely and reliably applied to humanitarian assistance.

### Next steps

The results from this study show a wide array of ethical issues that should be addressed when processing data in a humanitarian context. However, to our knowledge, to date no humanitarian data protection guidance is sufficiently comprehensive to provide practical guidance for all concerns identified in the literature. The ethical issues identified in this review should be used to inform the development of ethical codes of conduct (whether voluntary or mandated by organizations). Further, companies and institutions behind the various technologies—as well as the humanitarian organizations that use them to process data as part of their work—should investigate to what extent these ethical issues are being addressed, and where more needs to be done. Such guides should complement the practical and operational documents produced by OCHA [10], the IASC [35], or the ICRC [36]. Protection Information Management has produced principles and various training products that address both practical implementation needs as well as the underlying ethical considerations guiding them [132]. However, existing humanitarian data protection guidance and mechanisms are not sufficient to address all concerns identified in the literature and in this study. Likewise, training and accountability mechanisms to monitor the actual harm or potential for causing harm and to limit risks, are insufficient. These guidelines and mechanisms will need to be reviewed, expanded and informed by regular reviews that keep pace with technological change and changes in practice. Further research, especially using empirical methods, is necessary to better identify and understand the type and prevalence of ethical issues in the field.

Our findings indicate that more investigations are needed into the appropriate and inappropriate use of commonplace humanitarian tools and data management processes, such as CAPI, spreadsheets, filesharing, or use of online databases.

More research will be needed in the future focusing on ethical issues that are unique to conflict settings [e.g., 133, 134], as data processing without appropriate consideration of ethical issues in these settings arguably has the potential to cause far greater harm.

Finally, case studies of early adoptions of AI by humanitarian organizations should address which ethical considerations were given when using tools that may involve data processing using multiple services and companies globally, in order to inform local decisions. A useful example is Aiken et al. [135] who measured the biases and shortcomings of using machine learning for targeted aid distribution. Such research is urgently needed to create better guidance, training, and auditing methods to support humanitarian organizations to use data processing technologies as ethically as possible.

### Limitations of the scoping review

This study is limited to literature published from January 2010 through August 2024, and it includes only work from peer-reviewed sources. As mentioned above, identifying all relevant studies was a significant challenge due to the lack of a shared nomenclature across disciplines for humanitarian assistance, ethical issues, and data processing. As a result, potentially relevant articles that met the inclusion criteria may have been missed. Nonetheless, we believe that our search strategy represents the most comprehensive and inclusive set of keywords to capture studies in the diverse field of humanitarian assistance to date. The criteria and definitions selected for this scoping review allowed for a broader scope, enabling the inclusion of articles that may not explicitly label a setting as “humanitarian” but involve contexts aligning with our definition of a humanitarian crisis (e.g., natural hazards in low-income countries). This approach enhances definitional and conceptual clarity, offering a replicable framework to improve the consistency and relevance of future scoping studies.

By including only peer-reviewed publications this scoping review excludes the grey literature on humanitarian data processing and ethical concerns, which may skew the findings to some degree. Specifically, this review might include a slightly higher proportion of outdated data processing technologies or more abstract ethical concerns than it would if the grey literature had been incorporated. Peer-reviewed studies are often published at a slower pace than the grey literature, so this article may include a smaller proportion of cutting-edge technologies; likewise, the academic literature might privilege more abstract ethical topics, such as state surveillance using humanitarian data, compared to the applied or practical ethical concerns typically favored in the policy literature, such as ensuring data is accurate enough to inform assistance. Since the two literatures are in dialogue with one another, however, we do not expect findings to be significantly skewed. To these authors’ knowledge, a scoping review combining the peer-reviewed and grey literatures has not been completed and remains a useful next step to advance the field.

As suggested by the Arksey and O’Malley framework, a consultation exercise with humanitarian and ethics experts will be organized to present our results, aid knowledge translation, ensure that the results from this study are relevant, and frame a future research agenda. The results of this consultation will be published separately.

## Conclusions

This extensive review of the literature highlights a growing concern over ethical challenges in data processing within humanitarian contexts, including those related to the increasing use of AI. Our findings underscore significant ethical risks associated with data processing in these settings, including potential harm, lack of direct benefits, infringement on populations’ autonomy, and the unfair allocation of resources. Notably, nearly one in three of the studies reviewed address AI technologies, with the primary ethical concern being biased data processing leading to inequitable distribution of humanitarian assistance. Unreliable data and privacy, especially regarding the inadvertent sharing of sensitive data with third parties, were also common AI-related ethical concerns. 

The underrepresentation of perspectives from low and middle-income countries in the academic discourse further exacerbates these challenges, highlighting the urgent need for more diverse and inclusive perspectives. Additional research, especially using empirical methods, is necessary to better identify and understand the type and prevalence of ethical issues in the field. While disasters predominate the literature, more studies are needed to investigate the unique ethical issues that arise in conflict settings to better address the heightened security risks to vulnerable people in war.

Existing humanitarian data protection guidance as well as training and accountability methods for monitoring potential harm and to limit risks are insufficient to address all concerns identified in the literature and in this study. These guidelines and mechanisms will need to be reviewed, expanded, and informed by regular reviews that keep pace with technological change and changes in practice. Likewise, companies and institutions behind the various technologies—as well as the humanitarian organizations that use them to process data as part of their work—should investigate to what extent the ethical issues identified in this study are being addressed, and where more needs to be done.

Finally, investigations are urgently needed into early adoptions of AI tools in humanitarian contexts, including the rapid spread of large language models such as ChatGPT, to ensure these technologies are harnessed with utmost ethical rigor, safeguarding the dignity and rights of those in crisis while enhancing the efficacy and fairness of humanitarian responses.

## Electronic supplementary material

Below is the link to the electronic supplementary material.

Supplementary Material 1

Supplementary Material 2

Supplementary Material 3

Supplementary Material 4

Supplementary Material 5

## Data Availability

All data generated or analyzed during this study are included in this published article and its supplementary information files.

## Abbreviations

AI: Artificial Intelligence
CAPI: Computer-assisted personal interviewing
COVID-19: Coronavirus disease (2019)
GDPR: General Data Protection Regulation
GIS: Geographic information system
IASC: Inter-Agency Standing Committee
ICRC: International Committee of the Red Cross
ICT: Information and communication technology
OCHA: United Nations Office for the Coordination of Humanitarian Affairs
PRISMA-ScR: Preferred Reporting Items for Systematic reviews and Meta-Analyses extension for Scoping Reviews
SMS: Short messaging service
UAV: Unmanned aerial vehicles
UNHCR: United Nations High Commissioner for Refugees
