# Supplementary material for: Ethical implications related to processing of personal data and artificial intelligence in humanitarian crises: a scoping review
Source: BMC Med Ethics. 2025 Apr 15;26:49. doi: 10.1186/s12910-025-01189-2 (PMC11998222; doi:10.1186/s12910-025-01189-2)
Supplement: Supplementary file 1 — Supplementary Material 1 [file 12910_2025_1189_MOESM1_ESM.docx]

**Appendix B**: Scoping Review Protocol Developed A Priori

This study uses the scoping review method established by Arksey and O’Malley [1] and further refined by Levac et al. [2] and follows the framework maintained by the Joanna Briggs Institute [3, 4]. The protocol was revised based on feedback received from the research team and incorporated the results from a pilot conducted for this study November-December 2019. It follows the Preferred Reporting Items for Systematic Reviews and Meta-analysis for Protocols – Extension for Scoping Reviews (PRISMA-ScR) [5] for complete and transparent publishing of scoping reviews. This protocol has been adapted from Tricco et al. [6].

| **Review title and timescale** | |
| --- | --- |
| 1 | **Review title:** |
|  | Ethical Implications Related to Processing Personal Data in Humanitarian Crises: A Scoping Review |
| 2 | **Anticipated or actual start date:** |
|  | 12/2/2019 |
| 3 | **Anticipated completion date:** |
|  | 8/1/2020 |
| 4 | **Stage of review at time of this submission:** |
|  | \| **Review stage** \| **Started** \| **Completed** \| \| --- \| --- \| --- \| \| Preliminary searches \| ☐ \| X \| \| Piloting of the study selection process \| ☐ \| X \| \| Formal screening of search results against eligibility criteria \| X \| ☐ \| \| Data extraction \| ☐ \| ☐ \| \| Risk of bias (quality) assessment \| ☐ \| N/A \| \| Data analysis \| ☐ \| ☐ \| |
|  | |
| **Review team details** | |
| 5 | **Named contact** |
|  | Tino Kreutzer |
| 6 | **Named contact email** |
|  | kreutzer@yorku.ca |
| 7 | **Named contact address** |
|  | Suite 2150, Dahdaleh Building, York University  88 The Pond Road, Toronto, Ontario, Canada, M3J 1P3 |
| 8 | **Named contact phone number** |
|  | +1 416-736-2100 x34447 |
| 9 | **Organizational affiliation of the review** |
|  | Dahdaleh Institute for Global Health Research, York University |
| 10 | **Review team members and their organizational affiliations** |
|  | Mr. Tino Kreutzer, York University (PhD candidate)  Dr. James Orbinski, York University (dissertation supervisor)  Dr. Lora Appel, York University (dissertation committee member)  Dr. Aijun An, York University (dissertation committee member)  Dr. Patrick Vinck, Harvard University (dissertation committee member) |
| 11 | **Funding sources/sponsors** |
|  | N/A |
| 12 | **Conflicts of interest** |
|  | Authors have no known conflicts of interest to declare. |
|  | |
| **Review methods** | |
| 13 | **Review question(s):** |
|  | The objective of this scoping review is to map existing research on ethical implications stemming from the processing of data to inform humanitarian assistance. The specific research questions are:  Which **ethical implications** related to processing data from people affected by humanitarian crises in order to inform humanitarian assistance have been raised?  **What was the empirical basis** used to establish these implications?  **Which technologies** did these implications relate to?  **What types of humanitarian crises** did the study relate to? |
| 14 | **Literature Search:** |
|  | Comprehensive literature searches of electronic databases were conducted on March 31, 2020 using Ovid, Ebsco, Web of Science, and Proquest to search 20 databases for relevant studies. Only studies published in English, French, or Spanish were included. Search was limited to publications from 2010 onwards. |
| 15 | **URL to search strategy:** |
|  | See <https://bit.ly/3fIqJgO> for full details and search syntax. |
| 16 | **Condition or domain being studied:** |
|  | Ethical implications stemming from the processing of data relating to people affected by a humanitarian crisis with the explicit goal or potential of informing humanitarian assistance. |
| 17 | **Population/Problem:** |
|  | People affected by a **humanitarian crisis**.^[[1]](#footnote-2)^ This includes armed conflicts, natural disasters, and large public health emergencies—as well as refugees and migrants fleeing from such a crisis, regardless of their current location. We will also include studies that concern humanitarian assistance (including related fields such as disaster response or emergency management) that are global in scope. The following are included for the purpose of this study:   - Natural disasters in low or lower middle-income countries (defined as countries that ranked low income or low middle income at least once by the World Bank between 2011 and 2020) [8] - The Ebola outbreaks in West Africa (2014-2016) and DR Congo (2018-2020) - Any events taking place in countries registered in the same year as recipients by UNOCHA’s Financial Tracking Service (https://fts.unocha.org)   Excluded are:   - Studies related solely on natural disasters high or high middle-income countries |

| 18 | **Intervention(s)/Exposure(s):** |
| --- | --- |
|  | Studies that **investigate the processing of data**^[[2]](#footnote-3)^ **relating to people affected by a humanitarian crisis** **with the explicit goal or potential of informing humanitarian assistance**^[[3]](#footnote-4)^.  Excluded from this definition are:   - Studies that discuss data processing purely for research purposes - Any technologies that do not collect or process data related to affected people, e.g., robotics for clearing debris or land mines or any other robotics - Military aircraft (unless used to collect such data to support humanitarian assistance) - Algorithmic models for predicting the occurrence or impacts of natural hazards - Tools used for planning humanitarian logistics as long as they do not involve personal data from affected people (e.g., relief / distribution networks, supply chain management, and resource scheduling). |
| 19 | **Comparator(s)/Control(s):** |
|  | Studies without a comparator are eligible for inclusion. Any comparator is relevant for inclusion, such as comparing different technologies with each other or versus no technology used. |
| 20 | **Types of study to be included:** |
|  | All study designs will be eligible for inclusion.  Excluded are:   - Non peer-reviewed studies - Congressional documents and publications (e.g., committee hearing reports) - Conference announcements or summaries (e.g., “abstract list”, “poster abstracts”, etc.) - Book reviews |
| 21 | **Context:** |
|  | For feasibility reasons, we restrict the review to studies published since 1/1/2010. Studies in all countries or territories affected by a humanitarian crisis (or relevant host countries for displaced populations) will be included, as defined in point 17. |
| 22 | **Primary outcome(s):** |
|  | Studies that **investigate ethical implications**^[[4]](#footnote-5)^ **stemming from the processing of such data** (as defined in point 18).  Only studies that contain a **meaningful discussion** about this subject will be included. This will be assessed initially by whether ethical issues are explicitly mentioned in the abstract (including by looking for a broad range of potential synonyms, such as challenges, problems, risks, etc.). During the full text review this will be assessed qualitatively by two reviewers.  Empirical studies and those developing theory or frameworks, as well as reports, commentary or other types of articles will be included. |
| 23 | **Secondary outcome(s):** |
|  | Not applicable. |
| 24 | **Data extraction (selection and coding):** |
|  | Study selection and coding will be done using the DistillerSR software. Using the a priori eligibility criteria (points 17, 18, 20, and 22), standardized questionnaires have been developed. Testing and training exercises will precede each level of screening. Reviewer pairs will screen references and full texts independently.  The following selection process will be used:  Title screening   - Two reviewers will independently select studies solely on their title and bibliographic information - Daily meetings will be held to compare working definitions and discuss rating discrepancies during the first 1,000 references being reviewed - Each reference may be excluded based on the following screening questions:   1. Is the study likely to be about a humanitarian crisis?   2. [if (1) = yes or unsure]: Is the study likely to investigate the processing of data relating to people affected by a humanitarian crisis?   3. Is the type of study and study language eligible? - A study is excluded if any question was answered ‘no’ - Any conflicts during this title screening will be included in the abstract review, meaning that if one reviewer included and one excluded the reference it is considered included   Abstract screening   - Two reviewers will independently select studies based on their title, abstract, and bibliographic details - Daily meetings will be held during the review of the first 100 references to discuss rating discrepancies - Each reference may be excluded based on the abovementioned title screening questions, plus the following:   1. [if (2) = yes or unsure]: Is the study likely to have a meaningful discussion of ethical implications? - A study is excluded if any question was answered ‘no’ - Any conflicts during this abstract screening will be included in the full text review, meaning that if one reviewer included and one excluded the reference it is considered included   Full text review   - Two reviewers will independently select studies based on the full text - Daily meetings will be held during the review of the first 20 references to discuss rating discrepancies - Each reference may be excluded based on the abovementioned screening criteria (see Appendix for the exact screening questions) - A study is excluded if any question was answered ‘no’ - Rating discrepancies will be resolved by discussion or by using a third adjudicator   Data extraction   - Two reviewers will independently collect data from each study. In case a large number of studies is identified (>30), we will conduct data extraction with one reviewer and one verifier. - Data collection form will be pilot-tested prior to full data extraction - We will extract data on   1. Study characteristics (e.g., country of corresponding author, journal discipline)   2. Population characteristics (e.g., type of humanitarian crisis)   3. intervention characteristics (e.g., purpose of data processing, technologies described)   4. Outcomes (e.g., specific ethical implications identified, whether implications are based on empirical data or theoretical, details of empirical data, whether artificial intelligence or related technologies were mentioned, specific implementation and/or compliance methods that may have been proposed) |
| 25 | **Risk of bias (quality) assessment:** |
|  | No quality appraisal will be conducted as this is a scoping review. This is consistent with the framework proposed by Arksey and O’Malley, as well as the Joanna Briggs Institute guidance on conducting Scoping Reviews. |
| 26 | **Strategy for data synthesis:** |
|  | Results will be summarized quantitatively (using frequencies) and qualitatively (using descriptive analytics) to map and to identify gaps in the existing literature. |
| 27 | **Analysis of subgroups or subsets:** |
|  | Not applicable |
|  |  |
| **Review general information** | |
| 28 | **Type of review** |
|  | Scoping review |
| 29 | **Language** |
|  | English |
| 30 | **Country** |
|  | Canada |
| 31 | **Other registration details** |
|  | Not applicable |
| 32 | **Reference and/or URL for published protocol** |
|  | Not applicable |
| 33 | **Dissemination plans:** |
|  | Do you intend to publish the review on completion?  Yes x No ☐ |
| 34 | **Keywords** |
|  |  |
| 35 | **Details of any existing review of the same topic by the same authors.** |
|  | Not applicable |
| 36 | **Current review status** |
|  | Ongoing (study selection phase) |
| 37 | **Any additional information:** |
|  | Not applicable |
| 38 | **Details of final report/publication(s):** |
|  | Not applicable (review still in progress) |

**References**

1. Arksey H, O'Malley L. Scoping studies: towards a methodological framework. Int J Soc Res Methodol. 2005;8:19-32.

2. Levac D, Colquhoun H, O'Brien KK. Scoping studies: advancing the methodology. Implement Sci. 2010;5:69.

3. Peters MDJ, Godfrey CM, Khalil H, McInerney P, Parker D, Soares CB. Guidance for conducting systematic scoping reviews. Int J Evid-Based Healthc. 2015;13:141-6.

4. Peters MDJ, Godfrey C, Mcinerney P, Baldini Soares C, Khalil H, Parker D. Chapter 11: Scoping reviews. In: Aromataris E, Munn Z, editors. Joana Briggs Inst Rev Man [Internet]. 2017. p. 6–24. Available from: https://reviewersmanual.joannabriggs.org/

5. Tricco AC, Lillie E, Zarin W, O'Brien KK, Colquhoun H, Levac D, et al. PRISMA extension for scoping reviews (PRISMA-ScR): checklist and explanation. Ann Intern Med. 2018;169:467-73.

6. Tricco AC, Zarin W, Rios P, Pham B, Straus SE, Langlois EV. Barriers, facilitators, strategies and outcomes to engaging policymakers, healthcare managers and policy analysts in knowledge synthesis: a scoping review protocol. BMJ Open. 2016;6:e013929.

7. WHO. Risk reduction and emergency preparedness. Geneva, Switzerland: World Health Organization; 2007.

8. World Bank. World Bank country and lending groups. Washington, D.C., United States: The World Bank; 2020.

9. OCHA. Data responsibility guidelines. Geneva: UN Office for the Coordination of Humanitarian Affairs; 2019.

10. Pictet J. The fundamental principles of the Red Cross. Geneva: ICRC; 1979.

11. IASC. Protection of internally displaced persons. Geneva: IASC; 1999.

12. Slim H. Humanitarian ethics: a guide to the morality of aid in war and disaster. Oxford: Oxford University Press; 2015.

1. Humanitarian crises are defined here as “an event or series of events representing a critical threat to the health, safety, security or wellbeing of a community, usually over a wide area” [7]. [↑](#footnote-ref-2)
2. Data processing is defined here as “Any operation or set of operations which is performed on data or on sets of data, whether or not by automated means, such as collecting, registering, storing, adapting or altering, cleaning, filing, retrieving, using, disseminating, transferring and retaining or destroying” [9]. [↑](#footnote-ref-3)
3. Humanitarian assistance refers to coordinated actions that save lives, alleviate suffering, and maintain human dignity during and after human-made crises and disasters caused by natural hazards [10]. Humanitarian assistance here is considered to include “protection”, which “encompasses all activities aimed at obtaining full respect for the rights of the individual in accordance with the letter and the spirit of the relevant bodies of law” [11]. [↑](#footnote-ref-4)
4. Ethical implications are defined here as actions that may not be conforming to moral standards, particularly those set out by various humanitarian principles [12] because of the risks they presented [↑](#footnote-ref-5)
