# Supplementary material for: Ethical implications related to processing of personal data and artificial intelligence in humanitarian crises: a scoping review
Source: BMC Med Ethics. 2025 Apr 15;26:49. doi: 10.1186/s12910-025-01189-2 (PMC11998222; doi:10.1186/s12910-025-01189-2)
Supplement: Supplementary file 2 — Supplementary Material 2 [file 12910_2025_1189_MOESM2_ESM.docx]

**Appendix E**: Results Table for Studies Discussing Artificial Intelligence (n = 66)

|  | | Count |
| --- | --- | --- |
| Year of publication | 2010 | 0 |
|  | 2012 | 0 |
|  | 2013 | 0 |
|  | 2014 | 3 |
|  | 2015 | 3 |
|  | 2016 | 4 |
|  | 2017 | 2 |
|  | 2018 | 5 |
|  | 2019 | 8 |
|  | 2020 | 8 |
|  | 2021 | 3 |
|  | 2022 | 8 |
|  | 2023 | 12 |
|  | 2024 | 10 |
| Type of humanitarian crisis | Disaster | 23 |
|  | Refugees or migrants who fled a humanitarian crisis | 18 |
|  | Armed conflict | 16 |
|  | Not specified | 15 |
| Purposes of data processing | Assessment (of needs, damage, etc.) | 20 |
|  | Delivery of assistance | 15 |
|  | Registration / case management | 15 |
|  | Forecasting / modeling / early warning | 14 |
|  | Medical care or public health | 8 |
|  | Other | 7 |
|  | Accountability (complaints, feedback collection, etc. ) | 6 |
|  | Human rights violations | 6 |
|  | Search and rescue | 6 |
|  | Logistics | 4 |
|  | Cash transfer | 2 |

|  |  |  |
| --- | --- | --- |
| Specific technologies described | AI / algorithms / machine learning | 66 |
|  | Big data | 29 |
|  | Social media | 28 |
|  | Biometrics | 15 |
|  | Satellite imagery | 14 |
|  | UAV | 13 |
|  | Medical data | 7 |
|  | Call data records | 6 |
|  | Cash distribution | 4 |
|  | Blockchain / distributed ledger technology | 4 |
|  | Data storage | 3 |
|  | Computer-assisted telephone interviewing (CATI) | 1 |

| Ethical Issues Discussed | | Count |
| --- | --- | --- |
| Autonomy | Lack of consent: Data is collected without informed consent | 25 |
|  | Lack of respect: People/communities are not treated with respect | 17 |
|  | Data agency: People do not have the right to control, access, or delete their data | 16 |
|  | Participation: People/communities are not involved in decisions to use of new/experimental technologies for collecting data | 14 |
|  | Undisclosed use: Data may be used beyond purposes for which they were collected | 12 |
|  | Autonomy: Unwillingness to share data does not lead to disadvantages (e.g., exclusion from assistance or protection | 12 |
|  | Lack of group agency: Processed information is not available to affected communities | 1 |
| Beneficence | Unreliability: Processed data is inaccurate and does not sufficiently reflect reality to inform assistance | 41 |
|  | Dependence: Data is processed with the assistance of a political, economic, or military entity | 30 |
|  | Ineffective or inefficient: Not producing expected result, unmet expectations | 21 |
|  | Non-neutrality: Data is processed in a way that benefits or appears to benefit one side of the conflict over the other | 20 |
|  | Lack of action: Processed data is not utilized to inform assistance to the affected person/community | 12 |

| Non-maleficence | Privacy: Personal/sensitive data is shared with third parties | 41 |
| --- | --- | --- |
|  | Harm: People suffer physical or psychological harm as a result of data processing | 38 |
|  | Power imbalance: Data processing reinforces or worsens a lack of power of affected people | 27 |
|  | Data security: Personal/sensitive data is not protected against malicious actors | 24 |
|  | Excess: More data was collected than necessary | 10 |
|  | Redress/rectification: People do not have the ability to correct wrong information about them or receive compensation | 5 |
| Justice | Bias: Data is processed in a way that may (dis)advantage some people disproportionate to their humanitarian needs | 48 |
|  | Unequal access to technology / exclusion from data collection | 24 |
|  | Lack of accountability: Endangering (or not protecting) rights; absolving responsibility | 21 |
|  | Unfair distribution of risks and benefits | 16 |
