# Supplementary material for: Ethical implications related to processing of personal data and artificial intelligence in humanitarian crises: a scoping review
Source: BMC Med Ethics. 2025 Apr 15;26:49. doi: 10.1186/s12910-025-01189-2 (PMC11998222; doi:10.1186/s12910-025-01189-2)
Supplement: Supplementary file 3 — Supplementary Material 3 [file 12910_2025_1189_MOESM3_ESM.docx]

# Appendix C: Search Strategy and Keywords Used for Each Database

| Concept |  | OVID | Ebsco | Scopus | WOS | Proquest |
| --- | --- | --- | --- | --- | --- | --- |
|  |  |  | (( | (TITLE-ABS-KEY ( | (TS=( | (noft( |
| Humanitarian assistance | 1 | humanitarian*.tw. | TI humanitarian* or AB humanitarian* or SU humanitarian* | humanitarian* | humanitarian* | humanitarian* |
|  | 2 | relief work.tw. | or TI "relief work" or AB "relief work" or SU "relief work" | or "relief work" | or "relief work" | or "relief work" |
|  | 3 | aid work.tw. | or TI "aid work" or AB "aid work" or SU "aid work" | or "aid work" | or "aid work" | or "aid work" |
|  | 4 | (disaster? adj (relief or response? or assistance)).tw. | or TI (disaster# N0 (relief or response$ or assistance)) or AB (disaster# N0 (relief or response$ or assistance)) or SU (disaster# N0 (relief or response$ or assistance)) | or (disaster# N/0 (relief or response or assistance)) | or (disaster$ NEAR/0 (relief or response or assistance)) | or (disaster? NEAR/0 (relief or response$ or assistance)) |
|  | 5 | emergency relief.tw. | or TI "emergency relief" or AB "emergency relief" | or "emergency relief" | or "emergency relief" | or "emergency relief" |
|  | 6 | ((conflict? or war?) adj10 (human rights or public health)).tw. | or TI ((conflict# or war#) N10 ("human rights" or "public health")) or AB ((conflict# or war#) N10 ("human rights" or "public health")) | or ((conflict# or war#) N/10 ("human rights" or "public health")) | or ((conflict$ or war$) NEAR/10 ("human rights" or "public health")) | or ((conflict? or war?) NEAR/10 ("human rights" or "public health")) |
|  | 7 | (ebola adj6 (west africa or sierra leone or liberia or guinea or 2014 or 2013)).tw. | or TI (ebola N6 ("west africa" or "sierra leone" or liberia or guinea or 2014 or 2013)) or AB (ebola N6 ("west africa" or "sierra leone" or liberia or guinea or 2014 or 2013)) | or (ebola N/6 ("west africa" or "sierra leone" or liberia or guinea or 2014 or 2013)) | or (ebola NEAR/6 ("west africa" or "sierra leone" or liberia or guinea or 2014 or 2013)) | or (ebola NEAR/6 ("west africa" or "sierra leone" or liberia or guinea or 2014 or 2013)) |
|  | 8 | acute malnutrition.tw. | or TI "acute malnutrition" or AB "acute malnutrition" | or "acute malnutrition" | or "acute malnutrition" | or "acute malnutrition" |
|  | 9 | (refugee* adj2 (camp* or assistance or population?)).tw. | or TI (refugee* N2 (camp* or assistance or population#)) or AB (refugee* N2 (camp* or assistance or population#)) | or (refugee* N/2 (camp* or assistance or population#)) | or (refugee* NEAR/2 (camp* or assistance or population$)) | or (refugee* NEAR/2 (camp* or assistance or population?)) |
|  | 10 | (displace* adj2 (forced or forcibly or population? or human? or internal*)).tw. | or TI (displace* N2 (forced or forcibly or population# or human# or internal*)) or AB (displace* N2 (forced or forcibly or population# or human# or internal*)) | or (displace* N/2 (forced or forcibly or population# or human# or internal*)) | or (displace* NEAR/2 (forced or forcibly or population$ or human$ or internal*)) | or (displace* NEAR/2 (forced or forcibly or population? or human? or internal*)) |
|  | 11 | (((population? or person* or communit*) adj3 affected) adj1 (conflict? or violence)).tw. | or TI (((population# or person* or communit*) N3 affected) N1 (conflict# or violence)) or AB (((population# or person* or communit*) N3 affected) N1 (conflict# or violence)) | or (((population# or person* or communit*) N/3 affected) N/1 (conflict# or violence)) | or (((population$ or person* or communit*) NEAR/3 affected) NEAR/1 (conflict$ or violence)) | or (((population? or person* or communit*) NEAR/3 affected) NEAR/1 (conflict? or violence)) |
|  | 12 | or/ 1-11 | ) | ) | ) | ) |
|  |  |  | or ( | or ( | or (TS= | or (( |
|  | 13 | (cris?s or emergenc* or disaster? or "natural hazard?").tw. | (TI (cris#s or emergenc* or disaster# or "natural hazard#") or AB (cris#s or emergenc* or disaster# or "natural hazard#") | TITLE-ABS-KEY(cris#s or emergenc* or disaster#) | TITLE-ABS-KEY(cris#s or emergenc* or disaster# or "natural hazard#") | noft(cris?s or emergenc* or disaster? or "natural hazard?") |
|  |  |  | ) and | and | and | and |
|  | 14 | humanitarian*.af. | TX humanitarian* | ALL(humanitarian*) | ALL=humanitarian* | ft(humanitarian* |
|  | 15 | 13 and 14 | )) and | )) and | )) and | )))) and |
|  | 16 | 12 or 15 |  |  |  |  |
| ICT for data collection | 17 | ict.tw. | (TI ict or AB ict | TITLE-ABS-KEY(ict | TS=(ict | noft(ict |
|  | 18 | technolog*.tw. | or TI technolog* or AB technolog* | or technolog* | or technolog* | or technolog* |
|  | 19 | ((data or information) adj2 (system* or manage* or collection or analys?s or process*)).tw. | or TI ((data or information) N2 (system* or manage* or collection or analys#s or process*)) or AB ((data or information) N2 (system* or manage* or collection or analys#s or process*)) | or ((data or information) N/2 (system* or manage* or collection or analys#s or process*)) | or ((data or information) NEAR/2 (system* or manage* or collection or analys$s or process*)) | or ((data or information) NEAR/2 (system* or manage* or collection or analys?s or process*)) |
|  | 20 | (blockchain or distributed ledger).tw. | or TI (blockchain or "distributed ledger") or AB (blockchain or "distributed ledger") | or (blockchain or "distributed ledger") | or (blockchain or "distributed ledger") | or (blockchain or "distributed ledger") |
|  | 21 | (ai or artificial intelligence or machine learning or algorithm*).tw. | or TI (ai or "artificial intelligence" or "machine learning" or algorithm*) or AB (ai or "artificial intelligence" or "machine learning" or algorithm*) | or (ai or "artificial intelligence" or "machine learning" or algorithm*) | or (ai or "artificial intelligence" or "machine learning" or algorithm*) | or (ai or "artificial intelligence" or "machine learning" or algorithm*) |
|  | 22 | biometric*.tw. | or TI biometric* or AB biometric* | or biometric* | or biometric* | or biometric* |
|  | 23 | smartphone app*.tw. | or TI "smartphone app*" or AB "smartphone app*" | or "smartphone app*" | or "smartphone app*" | or "smartphone app*" |
|  | 24 | remote sensing.tw. | or TI "remote sensing" or AB "remote sensing" | or "remote sensing" | or "remote sensing" | or "remote sensing" |
|  | 25 | analytics.tw. | or TI analytics or AB analytics | or analytics | or analytics | or analytics |
|  | 26 | digital*.tw. | or TI digital* or AB digital* | or digital* | or digital* | or digital* |
|  | 27 | experimentation.tw. | or TI experimentation or AB experimentation | or experimentation | or experimentation | or experimentation |
|  | 28 | automat*.tw. | or TI automat* or AB automat* | or automat* | or automat* | or automat* |
|  | 29 | innovation?.tw. | or TI innovation# or AB innovation# | or innovation# | or innovation$ | or innovation? |
|  | 30 | remote management.tw. | or TI "remote management" or AB "remote management" | or "remote management" | or "remote management" | or "remote management" |
|  | 31 | cyber.tw. | or TI cyber or AB cyber | or cyber | or cyber | or cyber |
|  | 32 | big data.tw. | or TI "big data" or AB "big data" | or "big data" | or "big data" | or "big data" |
|  | 33 | (sms or text messag* or interactive voice recognition or online survey*).tw. | or TI (sms or "text messag*" or "interactive voice recognition" or "online survey*") or AB (sms or "text messag*" or "interactive voice recognition" or "online survey*") | or (sms or "text messag*" or "interactive voice recognition" or "online survey*") | or (sms or "text messag*" or "interactive voice recognition" or "online survey*") | or (sms or "text messag*" or "interactive voice recognition" or "online survey*") |
|  | 34 | (kobotoolbox or kobo or odk or open data kit).tw. | or TI (kobotoolbox or kobo or odk or "open data kit") or AB (kobotoolbox or kobo or odk or "open data kit") | or (kobotoolbox or kobo or odk or "open data kit") | or (kobotoolbox or kobo or odk or "open data kit") | or (kobotoolbox or kobo or odk or "open data kit") |
|  | 35 | crowdsourc*.tw. | or TI crowdsourc* or AB crowdsourc* | | or crowdsourc* | or crowdsourc* |
|  | 36 | social media.tw. | or TI "social media" or AB "social media" | | or "social media" | or "social media" |
|  | 37 | crisis adj (informatics or data or map*).tw. | or TI "crisis N0 (informatics or data or map*)" or AB "crisis N0 (informatics or data or map*)" | | or "crisis NEAR/0 (informatics or data or map*)" | or "crisis NEAR/0 (informatics or data or map*)" |
|  | 38 | digiti?ation.tw. | or TI digiti#ation or AB digiti#ation | | or digiti$ation | or digiti?ation |
|  | 39 | datafication.tw. | or TI datafication or AB datafication | | or datafication | or datafication |
|  | 40 | or/ 17-39 | ) | ) | ) | ) |
|  |  |  | and | and | and | and |
| Ethical concerns | 41 | concern?.tw. | (TI concern# or AB concern# | TITLE-ABS-KEY(concern# | TS=(concern$ | noft(concern? |
|  | 42 | risk?.tw. | or TI risk# or AB risk# | or risk# | or risk$ | or risk? |
|  | 43 | challenge?.tw. | or TI challenge# or AB challenge# | or challenge# | or challenge$ | or challenge? |
|  | 44 | harm?.tw. | or TI harm# or AB harm# | or harm# | or harm$ | or harm? |
|  | 45 | privacy.tw. | or TI privacy or AB privacy | or privacy | or privacy | or privacy |
|  | 46 | protection?.tw. | or TI protection# or AB protection# | or protection# | or protection$ | or protection? |
|  | 47 | humanitarian adj (principle? or standard? or guideline?).tw. | or TI humanitarian N0 (principle# or standard# or guideline#) or AB humanitarian N0 (principle# or standard# or guideline#) | or humanitarian N/0 (principle# or standard# or guideline#) | or humanitarian NEAR/0 (principle$ or standard$ or guideline$) | or humanitarian NEAR/0 (principle? or standard? or guideline?) |
|  | 48 | problem?.tw. | or TI problem# or AB problem# | or problem# | or problem$ | or problem? |
|  | 49 | bias?.tw. | or TI bias# or AB bias# | or bias# | or bias$ | or bias? |
|  | 50 | ethic*.tw. | or TI ethic* or AB ethic* | or ethic* | or ethic* | or ethic* |
|  | 51 | consequence?.tw. | or TI consequence# or AB consequence# | | or consequence$ | or consequence? |
|  | 52 | critique?.tw. | or TI critique# or AB critique# | | or critique$ | or critique? |
|  | 53 | insecurity.tw. | or TI insecurity or AB insecurity | | or insecurity | or insecurity |
|  | 54 | implications.tw. | or TI implications or AB implications | | or implications | or implications |
|  | 55 | peril?.tw. | or TI peril# or AB peril# | | or peril$ | or peril? |
|  | 56 | impact?.tw. | or TI impact# or AB impact# | | or impact$ | or impact? |
|  | 57 | or/ 41-56 | ) | ) | ) | ) and pd(2010-2019) |
|  |  |  | ) |  |  |  |
|  |  | 16 and 40 and 57 |  |  |  |  |
