# Supplementary material for: Ethical implications related to processing of personal data and artificial intelligence in humanitarian crises: a scoping review
Source: BMC Med Ethics. 2025 Apr 15;26:49. doi: 10.1186/s12910-025-01189-2 (PMC11998222; doi:10.1186/s12910-025-01189-2)
Supplement: Supplementary file 5 — Supplementary Material 5 [file 12910_2025_1189_MOESM5_ESM.docx]

# Appendix D: List of Studies Included in Scoping Review Results

1. Abbasi A, Dillon R, Rao HR, Liu Sheng OR. Preparedness and Response in the Century of Disasters: Overview of Information Systems Research Frontiers. Information Systems Research. 2024;35(2):460–8. doi:10.1287/isre.2024.intro.v35.n2
2. Açιkyιldιz Ç. ‘I know you like the back of my hand’: Biometric practices of humanitarian organisations in international aid. Disasters. 2023;48(2). doi:10.1111/disa.12612
3. Ahmad N. Refugees and Algorithmic Humanitarianism: Applying Artificial Intelligence To RSD Procedures and Immigration Decisions and Making Global Human Rights Obligations Relevant To AI Governance. International Journal on Minority and Group Rights. 2020;1–69. doi:10.1163/15718115-bja10007
4. Aiken E, Bellue S, Karlan D, Udry C, Blumenstock JE. Machine learning and phone data can improve targeting of humanitarian aid. Nature. 2022;603(7903):864–70. doi:10.1038/s41586-022-04484-9
5. Aiken E, Rolf E, Blumenstock J. Fairness and Representation in Satellite-Based Poverty Maps: Evidence of Urban-Rural Disparities and Their Impacts on Downstream Policy. Proceedings of the Thirty-Second International Joint Conference on Artificial Intelligence. 2023;5888–96. doi:10.24963/ijcai.2023/653
6. Algiriyage N, Prasanna R, Stock K, Doyle EE, Johnston D. Multi-source Multimodal Data and Deep Learning for Disaster Response: A Systematic Review. SN Computer Science. 2022;3(1). doi:10.1007/s42979-021-00971-4
7. Alvarado Garcia A, Wong-Villacres M, Miceli M, Hernández B, Le Dantec CA. Mobilizing Social Media Data: Reflections of a Researcher Mediating between Data and Organization. Proceedings of the 2023 CHI Conference on Human Factors in Computing Systems. 2023;1–19. doi:10.1145/3544548.3580916
8. Aziz A. Power geometries of mediated care: (Re)mapping transnational families and immobility of the Rohingya diaspora in a digital age. Media, Culture &Society. 2022;44(5):967–85. doi:10.1177/01634437211065690
9. Baharmand H, Saeed N, Comes T, Lauras M. Developing a framework for designing humanitarian blockchain projects. Computers in Industry. 2021;131:103487. doi:10.1016/j.compind.2021.103487
10. Baykurt B, Lyamuya A. Making up the predictable border: How bureaucracies legitimate data science techniques. New Media & Society. 2023;26(12):6958–74. doi:10.1177/14614448231161276
11. Beduschi A (2019) Digital identity: Contemporary challenges for data protection, privacy and non-discrimination rights. Big Data Soc 6:1–6
12. Beduschi A. Harnessing the potential of artificial intelligence for humanitarian action: Opportunities and risks. International Review of the Red Cross. 2022;104(919):1149–69. doi:10.1017/s1816383122000261
13. Bell D, Lycett M, Marshan A, Monaghan A. Exploring future challenges for big data in the humanitarian domain. Journal of Business Research. 2021;131:453–68. doi:10.1016/j.jbusres.2020.09.035
14. Belliveau J (2016) Humanitarian access and technology: Opportunities and applications. Procedia Eng 159:300–306
15. Bempong NE, De Castañeda RR, Schütte S, Bolon I, Keiser O, Escher G, Flahault A (2019) Precision Global Health - The case of Ebola: A scoping review. J Glob Health. https://doi.org/10.7189/jogh.09.010404
16. Bennett C (2014) Who knows who we are? Questioning DNA analysis in disaster victim identification. New Genet Soc 33:239–256
17. Benson J, Brand T, Christianson L, Lakeberg M. Localisation of digital health tools used by displaced populations in low and middle-income settings: a scoping review and critical analysis of the Participation Revolution. Conflict and Health. 2023;17(1). doi:10.1186/s13031-023-00518-9
18. Benson J, Lakeberg M, Brand T. Exploring the perspectives and practices of humanitarian actors towards the Participation Revolution in humanitarian digital health responses: a qualitative study. Globalization and Health. 2024;20(1). doi:10.1186/s12992-024-01042-y
19. Bernard R, Bowsher G, Milner C, Boyle P, Patel P, Sullivan R (2018) Intelligence and global health: assessing the role of open source and social media intelligence analysis in infectious disease outbreaks. J Public Heal 26:509–514
20. Bittner C, Glasze G, Turk C (2013) Tracing contingencies: analyzing the political in assemblages of web 2.0 cartographies. GeoJournal 78:935
21. Bock JG (2015) Firmer Footing for a Policy of Early Intervention: Conflict Early Warning and Early Response Comes of Age. J Inf Technol Polit 12:103–111
22. Bock JG (2016) Technology and Vulnerability in Early Warning: Ethical Use of IT in Dangerous Places. Inf Technol Dev 22:696–709
23. Boersma K, Büscher M, Fonio C. Crisis management, surveillance, and digital ethics in the COVID‐19 era. Journal of Contingencies and Crisis Management. 2022;30(1):2–9. doi:10.1111/1468-5973.12398
24. Brahimi L, Krishnaraj G, Pringle J, Schwartz L, O’Mathúna D, Hunt M. The Ethics of Humanitarian Innovation: Mapping Values Statements and Engaging with Value-Sensitive Design. Canadian Journal of Bioethics. 2023;6(2):1–10. doi:10.7202/1101123ar
25. Bravo RZB, Leiras A, Cyrino Oliveira FL (2019) The Use of UAVs in Humanitarian Relief: An Application of POMDP‐Based Methodology for Finding Victims. Prod Oper Manag 28:421–440
26. Brock PM, Mugera HK. Accelerating and enhancing the generation of socioeconomic data to inform forced displacement policy and response. Data & Policy. 2023;5. doi:10.1017/dap.2023.47
27. Brown C (2018) The use of ICTs in conflict and peacebuilding: A feminist analysis. Aust Fem Law J 44:137–153
28. Bruder M, Baar T. Innovation in humanitarian assistance—a systematic literature review. Journal of International Humanitarian Action. 2024;9(1). doi:10.1186/s41018-023-00144-3
29. Burger A, Oz T, Kennedy WG, Crooks AT (2019) Computational social science of disasters: Opportunities and challenges. Futur Internet 11:1–31
30. Burns R (2014) Moments of closure in the knowledge politics of digital humanitarianism. Geoforum 53:51–62
31. Burns R (2019) New Frontiers of Philanthro-capitalism: Digital Technologies and Humanitarianism. Antipode 51:1101–1122
32. Burns R. Rethinking big data in digital humanitarianism: Practices, epistemologies, and social relations. GeoJournal. 2015;80:477–90. doi:10.1007/s10708-014-9599-x
33. Burton J. “Doing no harm” in the digital age: What the digitalization of cash means for humanitarian action. International Review of the Red Cross. 2020;102(913):43–73. doi:10.1017/s1816383120000491
34. Canzutti L, Tazzioli M. Digital–Nondigital Assemblages: Data, Paper Trails, and Migrants’ Scattered Subjectivities at the Border. International Political Sociology. 2023;17(3). doi:10.1093/ips/olad014
35. Carenzo L, Barra FL, Ingrassia PL, Colombo D, Costa A, Corte F Della (2015) Disaster medicine through Google Glass. Eur J Emerg Med 22:222–225
36. Cenci A, Cawthorne D. Refining Value Sensitive Design: A (Capability-Based) Procedural Ethics Approach to Technological Design for Well-Being. Science and Engineering Ethics. 2020;26(5):2629–62. doi:10.1007/s11948-020-00223-3
37. Chan J, Bateman L, Olafsson G (2016) A people & purpose approach to humanitarian data information security and privacy. Procedia Eng 159:3–5
38. Cheesman M. Self-Sovereignty for Refugees? The Contested Horizons of Digital Identity. Geopolitics. 2020;27(1):134–59. doi:10.1080/14650045.2020.1823836
39. Cinnamon J, Jones SK, Adger WN (2016) Evidence and future potential of mobile phone data for disease disaster management. Geoforum 75:253–264
40. Clark N, Albris K. In the Interest(s) of Many: Governing Data in Crises. Politics and Governance. 2020 Dec 10;8(4):421–31. doi:10.17645/pag.v8i4.3110
41. Clark N, Guiffault F (2018) Seeing through the clouds: Processes and challenges for sharing geospatial data for disaster management in Haiti. Int J Disaster Risk Reduct 28:258–270
42. Clark NE, Chongtay R. Technological Mediation for Disaster Risk Management. Journal of Contingencies and Crisis Management. 2020;28(4):411–5. doi:10.1111/1468-5973.12331
43. Comes T (2016) Cognitive biases in humanitarian sensemaking and decision-making lessons from field research. 2016 IEEE Int Multi-Disciplinary Conf Cogn Methods Situat Aware Decis Support CogSIMA 2016 56–62
44. Comes T, Meesters K, Torjesen S (2019) Making sense of crises: the implications of information asymmetries for resilience and social justice in disaster-ridden communities. Sustain Resilient Infrastruct 4:124–136
45. Connolly D, Nam S, Goodman K. Solving old problems or making new ones? Blockchain technology for the protection of refugees and migrants. Journal of Human Rights. 2022;22(2):109–34. doi:10.1080/14754835.2022.2100984
46. Coppi G, Moreno Jimenez R, Kyriazi S. Explicability of humanitarian AI: a matter of principles. Journal of International Humanitarian Action. 2021;6(1). doi:10.1186/s41018-021-00096-6
47. Crawford K, Finn M (2015) The limits of crisis data: analytical and ethical challenges of using social and mobile data to understand disasters. GeoJournal 80:491–502
48. Curry T, Croitoru A, Crooks A, Stefanidis A (2019) Exodus 2.0: crowdsourcing geographical and social trails of mass migration. J Geogr Syst 21:161–187
49. Devidal P. Lost in digital translation? The humanitarian principles in the Digital age. International Review of the Red Cross. 2024;106(925):120–54. doi:10.1017/s1816383124000080
50. Devitt SK, Scholz J, Schless T, Lewis L. Developing a trusted human-AI network for humanitarian benefit. Digital War. 2023;4:1–17. doi:10.1057/s42984-023-00063-y
51. Dinh T, O’Leary S. The unaccounted effects of digital transformation: participatory accountability in a humanitarian organisation. Accounting, Auditing & Accountability Journal. 2024; doi:10.1108/aaaj-12-2022-6197
52. Dube T, Ncube SB, Mlotshwa S, Matsika GN, Maonde N. The changing face of monitoring and evaluation in the age of covid-19: Practitioners’ field experiences from Zimbabwe. Heliyon. 2021;7(6). doi:10.1016/j.heliyon.2021.e07386
53. Duffield M (2016) The resilience of the ruins: towards a critique of digital humanitarianism. Resilience 4:147–165
54. Easton C (2017) Analysing the Role of Privacy Impact Assessments in Technological Development for Crisis Management. J Contingencies Cris Manag 25:7–14
55. Egger C. The politics and spaces of public-private partnerships in humanitarian tech innovations. Environment and Planning C: Politics and Space. 2023;42(5):708–24. doi:10.1177/23996544231206822
56. Fast L (2017) Diverging Data: Exploring the Epistemologies of Data Collection and Use among Those Working on and in Conflict. Int Peacekeeping 24:706–732
57. Fast L, Read R. Using Data to Create Change? Interrogating the Role of Data in Ending Attacks on Healthcare. International Studies Review. 2022;24(3). doi:10.1093/isr/viac026
58. Fast L. Governing Data: Relationships, Trust & Ethics in leveraging Data & Technology in service of Humanitarian Health Delivery. Daedalus. 2023;152(2):125–40. doi:10.1162/daed_a_01996
59. Fayehun O, Akanle O, Popoola O, Okewumi E, Williams F, Adam A, et al. Data gathering and utilization: humanitarian targeting and ethical issues in northeastern Nigeria. Journal of International Humanitarian Action. 2023;8(1). doi:10.1186/s41018-023-00137-2
60. Fejerskov AM, Clausen M, Seddig S. Humanitarian ignorance: towards a new paradigm of non‐knowledge in Digital humanitarianism. Disasters. 2023;48(2). doi:10.1111/disa.12609
61. Franklinos LH, Parrish R, Burns R, Caflisch A, Mallick B, Rahman T, et al. Key opportunities and challenges for the use of big data in migration research and policy. UCL Open Environment. 2021;3. doi:10.14324/111.444/ucloe.000027
62. Garattini C, Raffle J, Aisyah DN, Sartain F, Kozlakidis Z (2019) Big Data Analytics, Infectious Diseases and Associated Ethical Impacts. Philos Technol 32:69–85
63. Gazi T, Gazis A. Humanitarian aid in the age of COVID-19: A review of big data crisis analytics and the General Data Protection Regulation. International Review of the Red Cross. 2020;102(913):75–94. doi:10.1017/s1816383121000084
64. Gazi T. Data to the rescue: how humanitarian aid NGOs should collect information based on the GDPR. Journal of International Humanitarian Action. 2020;5(1). doi:10.1186/s41018-020-00078-0
65. Gerdes A. A moderate interpretation of group privacy illustrated by cases from disaster management. Journal of Contingencies and Crisis Management. 2020;28(4):446–52. doi:10.1111/1468-5973.12336
66. Gorina Y, Redd JT, Hersey S, Jambai A, Meyer P, Kamara AS, et al. Ensuring ethical data access: the Sierra Leone Ebola Database (SLED) model. Annals of Epidemiology. 2020;46:1–4. doi:10.1016/j.annepidem.2020.04.001
67. Gueguen C, Snel N, Mutonji E. Turning mobile big data insights into public health responses in times of pandemics: Lessons learnt from the Democratic Republic of the Congo. Data & Policy. 2022;4. doi:10.1017/dap.2021.30
68. Guillén A, Teodoro E. Embedding Ethical Principles into AI Predictive Tools for Migration Management in Humanitarian Action. Social Sciences. 2023;12(2):53. doi:10.3390/socsci12020053
69. Gutierrez M, Bryant J. The Fading Gloss of Data Science: Towards an Agenda that Faces the Challenges of Big Data for Development and Humanitarian Action. Development. 2022;65(1):80–93. doi:10.1057/s41301-022-00327-2
70. Halkort M (2016) Liquefying Social Capital On the Bio-politics of Digital Circulation in a Palestinian Refugee Camp. Tecnoscienza-Italian J Sci Technol Stud 7:61–79
71. Halkort M (2019) Decolonizing data relations: On the moral economy of data sharing in Palestinian refugee camps. Can J Commun 44:317–329
72. Hart SU. From promise to practice: A cross-institutional analysis of design trends, enablers and challenges in blockchain-enabled cash and voucher delivery. International Journal of Disaster Risk Reduction. 2024;100:104129. doi:10.1016/j.ijdrr.2023.104129
73. Hassan NH, Rahim FA (2017) The rise of crowdsourcing using social media platforms: Security and privacy issues. Pertanika J Sci Technol 25:79–88
74. Haworth BT, Bruce E, Whittaker J, Read R (2018) The good, the bad, and the uncertain: Contributions of volunteered geographic information to community disaster resilience. Front Earth Sci 6:1–15
75. Hayes B (2017) Migration and data protection: Doing no harm in an age of mass displacement, mass surveillance and “big data.” Int Rev Red Cross 99:179
76. Hayes P, Jackson D. Care ethics and the responsible management of power and privacy in digitally enhanced disaster response. Journal of Information, Communication and Ethics in Society. 2020;18(1):157–74. doi:10.1108/jices-02-2019-0020
77. Hayes P, Kelly S (2018) Distributed morality, privacy, and social media in natural disaster response. Technol Soc 54:155–167
78. Henningsen G. Big Data for the Prediction of Forced Displacement. International Migration Review. 2023; doi:10.1177/01979183231195296
79. Herath T. The use of technology in global health humanitarian settings: how theory plays out in practice. Health and Technology. 2024;14(2):407–15. doi:10.1007/s12553-024-00817-y
80. Horowitz J. One Click from Conflict: Some Legal Considerations Related to Technology Companies Providing Digital Services in Situations of Armed Conflict. SSRN Electronic Journal. 2023; doi:10.2139/ssrn.4470988
81. Hudson LW (2013) SMS-based accountability to beneficiaries mechanisms in humanitarian aid and development. ACM Int Conf Proceeding Ser 2 NOTES:167–170
82. Hunt M, Pringle J, Christen M, Eckenwiler L, Schwartz L, Davé A (2016) Ethics of emergent information and communication technology applications in humanitarian medical assistance old. Int Health 8:239–245
83. Iazzolino G. Infrastructure of compassionate repression: making sense of biometrics in Kakuma refugee camp. Information Technology for Development. 2020;27(1):111–28. doi:10.1080/02681102.2020.1816881
84. Irom B, Gibbons S. From media to hypermedia: Journalistic representations of Rohingya refugees and humanitarian communication. Journalism. 2021;24(3):580–96. doi:10.1177/14648849211018591
85. Jacobsen KL (2010) Making design safe for citizens: a hidden history of humanitarian experimentation. Citizensh Stud 14:89
86. Jacobsen KL (2015) Experimentation in humanitarian locations: UNHCR and biometric registration of Afghan refugees. Secur Dialogue 46:144–164
87. Jacobsen KL (2017) On Humanitarian Refugee Biometrics and New Forms of Intervention. J Interv Statebuilding 11:529–551
88. Jacobsen KL, Fast L (2019) Rethinking access: how humanitarian technology governance blurs control and care. Disasters 43:S151–S168
89. Jacobsen KL, Sandvik KB (2018) UNHCR and the pursuit of international protection: accountability through technology? Third World Q 39:1508–1524
90. Jacobsen KL. Biometric data flows and unintended consequences of counterterrorism. International Review of the Red Cross. 2021 Apr;103(916–917):619–52. doi:10.1017/s1816383121000928
91. Jaljolie R, Dror T, Siriba DN, Dalyot S. Evaluating current ethical values of OpenStreetMap using value sensitive design. Geo-spatial Information Science. 2022;26(3):362–78. doi:10.1080/10095020.2022.2087048
92. Juntunen EA, Kalla C, Widera A, Hellingrath B. Digitalization potentials and limitations of cash-based assistance. International Journal of Disaster Risk Reduction. 2023;97:104005. doi:10.1016/j.ijdrr.2023.104005
93. Jutel O. Blockchain humanitarianism and crypto-colonialism. Patterns. 2022;3(1):100422. doi:10.1016/j.patter.2021.100422
94. Kinchin N, Mougouei D. What Can Artificial Intelligence Do for Refugee Status Determination? A Proposal for Removing Subjective Fear. International Journal of Refugee Law. 2022;34(3–4):373–97. doi:10.1093/ijrl/eeac040
95. Kozcuer C, Mollen A, Bießmann F. Towards Transnational Fairness in Machine Learning: A Case Study in Disaster Response Systems. Minds and Machines. 2024;34(2). doi:10.1007/s11023-024-09663-3
96. Kreutzer T, Vinck P, Pham PN, An A, Appel L, DeLuca E, et al. Improving humanitarian needs assessments through natural language processing. IBM Journal of Research and Development. 2020;64(1/2). doi:10.1147/jrd.2019.2947014
97. Krichen M, Abdalzaher MS. Advances in AI and Drone-based Natural Disaster Management: A Survey. 2023 20th ACS/IEEE International Conference on Computer Systems and Applications (AICCSA). 2023;1–6. doi:10.1109/aiccsa59173.2023.10479345
98. Kroener I, Barnard-Wills D, Muraszkiewicz J (2019) Agile ethics: an iterative and flexible approach to assessing ethical, legal and social issues in the agile development of crisis management information systems. Ethics Inf Technol 1
99. Kunz N (2019) An automated quantitative content analysis process for humanitarian logistics research. J Humanit Logist Supply Chain Manag 9:475–491
100. Lambert A (2016) Disaster data assemblages: five perspectives on social media and communities in response and recovery. Proc Annu Hawaii Int Conf Syst Sci 2016-March:2237–2245
101. Latif S, Qayyum A, Usama M, Qadir J, Zwitter A, Shahzad M (2019) Caveat Emptor: The Risks of Using Big Data for Human Development. IEEE Technol Soc Mag 38:82–90
102. Le Blond S, Cuevas A, Troncoso-Pastoriza JR, Jovanovic P, Ford B, Hubaux JP (2018) On Enforcing the Digital Immunity of a Large Humanitarian Organization. Proc - IEEE Symp Secur Priv 2018-May:424–440
103. Leasure DR, Kashyap R, Rampazzo F, Dooley CA, Elbers B, Bondarenko M, et al. Nowcasting Daily Population Displacement in Ukraine through Social Media Advertising Data. Population and Development Review. 2023;49(2):231–54. doi:10.1111/padr.12558
104. Lemieux V, Gallant A, Pourmalek P, Hamouda H, Johnston N, El-Ghazal S, et al. Designing recordkeeping systems for transitional justice and peace: ‘on the ground’ experiences and practices relating to organizations supporting conflict-affected peoples. Archival Science. 2024 Apr 22;24(2):227–55. doi:10.1007/s10502-024-09439-9
105. Lentz EC, Maxwell D. How do information problems constrain anticipating, mitigating, and responding to crises? International Journal of Disaster Risk Reduction. 2022;81:103242. doi:10.1016/j.ijdrr.2022.103242
106. Lev Aretz Y (2019) Data Philanthropy. SSRN Electron J. https://doi.org/10.2139/ssrn.3320798
107. Longboan L (2018) “I don’t want trouble”: Freedom of expression and use of backchannels in the wake of Typhoon Haiyan. Disaster Prev Manag An Int J 27:380–392
108. Loukinas P. Drones for Border Surveillance: Multipurpose Use, Uncertainty and Challenges at EU Borders. Geopolitics. 2021;27(1):89–112. doi:10.1080/14650045.2021.1929182
109. Lyamuya A. Humanitarian Innovation in Forced Displacement. International Journal of Communication. 2024;18:769–76.
110. Madianou M (2015) Digital Inequality and Second-Order Disasters: Social Media in the Typhoon Haiyan Recovery. Soc Media Soc. https://doi.org/10.1177/2056305115603386
111. Madianou M (2019) The Biometric Assemblage: Surveillance, Experimentation, Profit, and the Measuring of Refugee Bodies. Telev New Media 20:581–599
112. Madianou M, Ong JC, Longboan L, Cornelio JS (2016) The Appearance of Accountability: Communication Technologies and Power Asymmetries in Humanitarian Aid and Disaster Recovery. J Commun 66:960–981
113. Madianou M. Nonhuman humanitarianism: when “AI for good” can be harmful. Information, Communication &Society. 2021;24(6):850–68. doi:10.1080/1369118x.2021.1909100
114. Madon S, Schoemaker E (2019) Reimagining Refugee Identity Systems: A Sociological Approach. Inf. Commun. Technol. Dev. Strength. Southern-Driven Coop. as a Catal. Ict4d, Pt I
115. Madon S, Schoemaker E. Digital identity as a platform for improving refugee management. Information Systems Journal. 2021;31(6):929–53. doi:10.1111/isj.12353
116. Maghsoudi A, Harpring R, Piotrowicz WD, Kedziora D. Digital technologies for cash and voucher assistance in disasters: A cross-case analysis of benefits and risks. International Journal of Disaster Risk Reduction. 2023;96:103827. doi:10.1016/j.ijdrr.2023.103827
117. Maitland C, Martin J-L, Bravo MG, Bertram A. A Qualitative Difference: Integrating Qualitative Data into Humanitarian Response Operations. Proceedings of the 2022 International Conference on Information and Communication Technologies and Development. 2022;1–18. doi:10.1145/3572334.3572398
118. Marino S. Digital Solidarity and Ethical Tech for Refugees: Why We Need to Care More and Code Less. International Journal of Communication. 2022;16:5538–52.
119. Martin A, Sharma G, Peter de Souza S, Taylor L, van Eerd B, McDonald SM, et al. Digitisation and Sovereignty in Humanitarian Space: Technologies, Territories and Tensions. Geopolitics. 2022;28(3):1362–97. doi:10.1080/14650045.2022.2047468
120. Martin A, Taylor L. Exclusion and inclusion in identification: regulation, displacement and data justice. Information Technology for Development. 2020;27(1):50–66. doi:10.1080/02681102.2020.1811943
121. Martin RS, Painho M (2019) Geospatial preparedness: Empirical study of the joint effort to provide geospatial support to disaster response. Trans GIS 23:481
122. Martin-Shields C (2013) The Technologists Dilemma: Ethical Challenges of Using Crowdsourcing Technology in Conflict and Disaster-Affected Regions. Georg J Int Aff 14:157–163
123. Martin-Shields C, Munir-Asen K. Do Information Communication Technologies (ICTs) Support Self-Reliance among Urban Refugees? Evidence from Kuala Lumpur and Penang, Malaysia. International Migration Review. 2022;58(1):69–93. doi:10.1177/01979183221139277
124. Masiero S. Dark side of IT: A misleading expression? The Electronic Journal of Information Systems in Developing Countries. 2023;90(1). doi:10.1002/isd2.12293
125. Masinde BK, Gevaert CM, Nagenborg MH, Zevenbergen JA. Group-Privacy Threats for Geodata in the Humanitarian Context. ISPRS International Journal of Geo-Information. 2023;12(10):393. doi:10.3390/ijgi12100393
126. Meier P, Munro R (2010) The Unprecedented Role of SMS in Disaster Response: Learning from Haiti. Part a Spec issue cyber Chall Threat Oppor a networked world 30:91–103
127. Mesmar S, Talhouk R, Akik C, et al (2016) The impact of digital technology on health of populations affected by humanitarian crises: Recent innovations and current gaps. J Public Health Policy 37:S167–S200
128. Molnar P. Technology on the margins: AI and global migration management from a human rights perspective. Cambridge International Law Journal. 2019;8(2):305–30. doi:10.4337/cilj.2019.02.07
129. Monich A, Holm-Nielsen PV, Raju E. The stagnation of innovation in humanitarian cash assistance. Journal of International Humanitarian Action. 2023 Mar 17;8(1). doi:10.1186/s41018-023-00136-3
130. Mulder F, Ferguson J, Groenewegen P, Boersma K, Wolbers J (2016) Questioning Big Data: Crowdsourcing crisis data towards an inclusive humanitarian response. Big Data Soc 3:1–13
131. Mulder F. Humanitarian Data Justice: A structural data justice lens on civic technologies in post‐earthquake Nepal. Journal of Contingencies and Crisis Management. 2020;28(4):432–45. doi:10.1111/1468-5973.12335
132. Nair S, Attal-Juncqua A, Reddy A, Sorrell EM, Standley CJ. Assessing barriers, opportunities and future directions in health information sharing in humanitarian contexts: a mixed-method study. BMJ Open. 2022;12(4). doi:10.1136/bmjopen-2021-053042
133. Nemer M, Khader YS, Alyahya MS, Pirlot de Corbion A, Sahay S, Abu-Rmeileh NM. Personal data governance and privacy in digital reproductive, maternal, newborn, and child health initiatives in Palestine and Jordan: a mapping exercise. Frontiers in Digital Health. 2023;5. doi:10.3389/fdgth.2023.1165692
134. Nussbaumer A, Pope A, Neville K. A framework for applying ethics‐by‐design to decision support systems for emergency management. Information Systems Journal. 2021;33(1):34–55. doi:10.1111/isj.12350
135. O’Reilly CF, Caffrey L, Jagoe C. Disability Data Collection in a Complex Humanitarian Organisation: Lessons from a Realist Evaluation. International Journal of Environmental Research and Public Health. 2021;18(19):10334. doi:10.3390/ijerph181910334
136. Owino B. Harmonising data systems for cash transfer programming in emergencies in Somalia. Journal of International Humanitarian Action. 2020;5(1). doi:10.1186/s41018-020-00077-1
137. Palmer L (2014) Ushahidi at the Google interface: critiquing the ‘geospatial visualization of testimony’. Contin J Media Cult Stud 28:342–356
138. Paragi B, Altamimi A. Caring control or controlling care? Double bind facilitated by biometrics between UNHCR and Syrian refugees in Jordan. Society and Economy. 2022;206–31. doi:10.1556/204.2021.00027
139. Paragi B. Digital4development? European data protection in the Global South. Third World Quarterly. 2020;42(2):254–73. doi:10.1080/01436597.2020.1811961
140. Paragi B. The Art of Screening: Reasonable Efforts and Measures at the Nexus of Aid Work and Counterterrorism. Surveillance & Society. 2024;22(2):138–59. doi:10.24908/ss.v22i2.15634
141. Paul S, Sosale S (2020) Witnessing a Disaster: Public Use of Digital Technologies in the 2015 South Indian Floods. Digit Journal 8:15–31
142. Paulus D, de Vries G, Janssen M, Van de Walle B. Reinforcing data bias in crisis information management: The case of the yemen humanitarian response. International Journal of Information Management. 2023;72:102663. doi:10.1016/j.ijinfomgt.2023.102663
143. Paulus D, Fathi R, Fiedrich F, de Walle BV, Comes T. On the Interplay of Data and Cognitive Bias in Crisis Information Management: An Exploratory Study on Epidemic Response. Information Systems Frontiers. 2022;26(2):391–415. doi:10.1007/s10796-022-10241-0
144. Pauu KT, Wu J, Fan Y, Pan Q, Maka M-‘i-V. Differential Privacy and Blockchain-Empowered Decentralized Graph Federated Learning-Enabled UAVs for Disaster Response. IEEE Internet of Things Journal. 2024;11(12):20930–47. doi:10.1109/jiot.2023.3332216
145. Peckham R, Sinha R (2017) Satellites and the New War on Infection: Tracking Ebola in West Africa. Geoforum 80:24
146. Perakslis ED (2018) Using digital health to enable ethical health research in conflict and other humanitarian settings. Confl Health 12:23
147. Perera S (2017) To Boldly Know: Knowledge, Peacekeeping and Remote Data Gathering in Conflict-Affected States. Int Peacekeeping 24:803–822
148. Pham PN, Vinck P (2012) Technology fusion and their implications for conflict early warning systems, public health, and human rights. Health Hum Rights 14:106–117
149. Phillips J (2015) Exploring the citizen-driven response to crisis in cyberspace, risk and the need for resilience. 2015 IEEE Canada Int Humanit Technol Conf IHTC 2015. https://doi.org/10.1109/IHTC.2015.7238051
150. Phillips J (2018) Risk in a digital age: understanding risk in virtual networks through digital response networks (DRNs). Int Dev Plan Rev 40:239–272
151. Pizzi M, Romanoff M, Engelhardt T. AI for humanitarian action: Human rights and ethics. International Review of the Red Cross. 2020;102(913):145–80. doi:10.1017/s1816383121000011
152. Poblet M, Kolieb J (2018) Responding to Human Rights Abuses in the Digital Era: New Tools, Old Challenges. Stanford J Int Law 54:259–283
153. Pond P (2016) The space between us: Twitter and crisis communication. Int J Disaster Resil Built Environ 7:40–48
154. Rabet R, Bagree E, Salam Z, Wahoush O. Barriers and facilitators to digital primary health Care Access in immigrant and refugee populations: a scoping review. Journal of Public Health. 2024; doi:10.1007/s10389-024-02193-3
155. Ray A, Bala PK (2020) Social media for improved process management in organizations during disasters. Knowl Process Manag 27:63–74
156. Read R (2016) Tensions in un Information Management: Security, Data and Human Rights Monitoring in Darfur, Sudan. J Hum Rights Pract 8:101–115
157. Read R, Taithe B, Mac Ginty R (2016) Data hubris? Humanitarian information systems and the mirage of technology. Third World Q 37:1314–1331
158. Relyea B, Wringe A, Afaneh O, Malamas I, Teodoro N, Ghafour M, et al. Stakeholders' Perspectives on the Challenges of Emergency Obstetric Referrals and the Feasibility and Acceptability of an mHealth Intervention in Northern Iraq. Frontiers in Global Women’s Health. 2021;2. doi:10.3389/fgwh.2021.662256
159. Resor E (2016) The Neo-Humanitarians: Assessing the Credibility of Organized Volunteer Crisis Mappers. Policy and Internet 8:34
160. Roberts T, Marchais G (2018) ASSESSING THE ROLE OF SOCIAL MEDIA AND DIGITAL TECHNOLOGY IN VIOLENCE REPORTING. Contemp Readings Law Soc Justice 10:9–42
161. Rocca R, Tamagnone N, Fekih S, Contla X, Rekabsaz N. Natural language processing for humanitarian action: Opportunities, challenges, and the path toward humanitarian NLP. Frontiers in Big Data. 2023;6. doi:10.3389/fdata.2023.1082787
162. Roth S, Luczak-Roesch M. Deconstructing the data life-cycle in digital humanitarianism. Information, Communication & Society. 2018;23(4):555–71. doi:10.1080/1369118x.2018.1521457
163. Rothe D, Fröhlich C, Rodriguez Lopez JM. Digital Humanitarianism and the Visual Politics of the Refugee Camp: (Un)Seeing Control. International Political Sociology. 2020;15(1):41–62. doi:10.1093/ips/olaa021
164. Sandvik KB (2016) The humanitarian cyberspace: shrinking space or an expanding frontier? Third World Q 37:17
165. Sandvik KB, Gabrielsen Jumbert M, Karlsrud J, Kaufmann M (2014) Humanitarian technology: A critical research agenda. Int Rev Red Cross 96:219–242
166. Sandvik KB, Jacobsen KL, McDonald SM (2017) Do no harm: A taxonomy of the challenges of humanitarian experimentation. Int Rev Red Cross 99:319–344
167. Sandvik KB, Lohne K (2014) The Rise of the Humanitarian Drone: Giving Content to an Emerging Concept. Millenn 43:145–164
168. Sandvik KB, Lohne K. The struggle against sexual violence in conflict: Investigating the digital turn. International Review of the Red Cross. 2020;102(913):95–115. doi:10.1017/s1816383121000060
169. Sandvik KB. Digital Refugee Lawyering: Risk, Legal Knowledge, and Accountability. Refugee Survey Quarterly. 2021;40(4):414–32. doi:10.1093/rsq/hdab013
170. Scarnecchia DP, Raymond NA, Greenwood F, Howarth C, Poole DE (2017) A Rights-based Approach to Information in Humanitarian Assistance. PLoS Curr 1–5
171. Schoemaker E, Baslan D, Pon B, Dell N. Identity at the margins: data justice and refugee experiences with Digital Identity Systems in Lebanon, Jordan, and Uganda. Information Technology for Development. 2020;27(1):13–36. doi:10.1080/02681102.2020.1785826
172. Schopper D, Dawson A, Upshur R, Ahmad A, Jesani A, Ravinetto R, Segelid MJ, Sheel S, Singh J (2015) Innovations in research ethics governance in humanitarian settings. BMC Med Ethics 16:1–12
173. Seff I, Vahedi L, McNelly S, Kormawa E, Stark L. Remote evaluations of violence against women and girls interventions: A rapid scoping review of tools, ethics and safety. BMJ Global Health. 2021;6(9). doi:10.1136/bmjgh-2021-006780
174. Sekara V, karsai M, moro E, kim D, delamonica E, cebrian M, et al. The Opportunities, Limitations, and Challenges in Using Machine Learning Technologies for Humanitarian Work and Development. Advances in Complex Systems. 2024;27(03). doi:10.1142/s0219525924400022
175. Seyedsayamdost E, Vanderwal P. From Good Governance to Governance for Good: Blockchain for Social Impact. Journal of International Development. 2020;32(6):943–60. doi:10.1002/jid.3485
176. Shah DC, Anderson C, Breimyer P, et al (2015) Application of graph methods for leveraging open source data during disaster response. Proc 5th IEEE Glob Humanit Technol Conf GHTC 2015 259–266
177. Shanley LA, Burns R, Bastian Z, Robson ES (2013) Tweeting up a storm: The promise and perils of crisis mapping. Photogramm Eng Remote Sensing 79:865–879
178. Sheather J, Jobanputra K, Schopper D, Pringle J, Venis S, Wong S, Vincent-Smith R (2016) A Médecins Sans Frontières Ethics Framework for Humanitarian Innovation. PLoS Med 13:1–10
179. Shemak A (2014) The cartographic dimensions of humanitarianism: Mapping refugee spaces in postearthquake Haiti. Cult Dyn 26:251–275
180. Shoemaker E, Kristinsdottir GS, Ahuja T, Baslan D, Pon B, Currion P, Gumisizira P, Dell N (2019) Identity at the margins: Examining refugee experiences with digital identity systems in Lebanon, Jordan, and Uganda. COMPASS 2019 - Proc 2019 Conf Comput Sustain Soc 206–217
181. Sithole TO. The use of digital platforms by citizen aid actors during the Ukraine humanitarian crisis. Frontiers in Human Dynamics. 2024;6. doi:10.3389/fhumd.2024.1326769
182. Sivanesan S (2019) Alex & I: narrative and network resistance. Soc Identities 25:559
183. Stys P, Muhindo S, N’simire S, Tchumisi I, Muzuri P, Balume B, et al. Reprint of: Trust, quality, and the Network Collection Experience: A tale of two studies on the Democratic Republic of the Congo. Social Networks. 2022;69:307–25. doi:10.1016/j.socnet.2022.01.003
184. Tajudeen R, Silaigwana B, Yavlinsky A, Edwards SJL. Research ethics during infectious disease outbreaks: A survey of African research stakeholders using the Ebola virus disease outbreak as a case. Journal of Public Health in Africa. 2023;14(9). doi:10.4081/jphia.2023.1632
185. Talbot R. Automating occupation: International humanitarian and human rights law implications of the deployment of facial recognition technologies in the occupied Palestinian territory. International Review of the Red Cross. 2020 Aug;102(914):823–49. doi:10.1017/s1816383121000746
186. Tamagnone N, Fekih S, Contla X, Orozco N, Rekabsaz N. Leveraging Domain Knowledge for Inclusive and Bias-aware Humanitarian Response Entry Classification. Proceedings of the Thirty-Second International Joint Conference on Artificial Intelligence. 2023;6219–27. doi:10.24963/ijcai.2023/690
187. Tanzi T, Apvrille L, Dugelay JL, Roudier Y (2014) UAVs for humanitarian missions: Autonomy and reliability. Proc 4th IEEE Glob Humanit Technol Conf GHTC 2014 271–278
188. Tanzi TJ, Chandra M, Isnard J, Camara D, Sebastien O, Harivelo F (2016) Towards “Drone-Borne” Disaster Management: Future Application Scenarios. ISPRS Ann Photogramm Remote Sens Spat Inf Sci III–8:181–189
189. Tatham P, Ball C, Wu Y, Diplas P (2017) Long-endurance remotely piloted aircraft systems (LE-RPAS) support for humanitarian logistic operations. J Humanit Logist Supply Chain Manag 7:2–25
190. Tatsidou E, Tsiamis C, Karamagioli E, Boudouris G, Pikoulis A, Kakalou E, Pikoulis E (2019) Reflecting upon the humanitarian use of unmanned aerial vehicles (drones). Swiss Med Wkly 149:1–6
191. Taylor L (2016) The ethics of big data as a public good: which public? Whose good? Philos Trans R Soc A Math Phys Eng Sci 374:20160126
192. Taylor L, Schroeder R (2015) Is bigger better? The emergence of big data as a tool for international development policy. GeoJournal 80:503–518
193. Tazzioli M. Digital expulsions: Refugees’ carcerality and the technological disruptions of asylum. Environment and Planning C: Politics and Space. 2023;41(7):1301–16. doi:10.1177/23996544231196680
194. Tazzioli M. Governing refugees through disorientation: Fragmented knowledges and forced technological mediations. Review of International Studies. 2022;48(3):425–40. doi:10.1017/s0260210522000079
195. Tedeschi C. Ethical, Legal, and Social Challenges in the Development and Implementation of Disaster Telemedicine. Disaster Medicine and Public Health Preparedness. 2020;15(5):649–56. doi:10.1017/dmp.2020.118
196. Thylin T, Duarte MFN (2019) Leveraging blockchain technology in humanitarian settings - opportunities and risks for women and girls. Gend Dev 27:317
197. Tran T, Valecha R, Rad P, Rao HR. An Investigation of Misinformation Harms Related to Social Media during Two Humanitarian Crises. Information Systems Frontiers. 2020;23(4):931–9. doi:10.1007/s10796-020-10088-3
198. Tullis JA, Kar B. Where Is the Provenance? Ethical Replicability and Reproducibility in GIScience and Its Critical Applications. Annals of the American Association of Geographers. 2020;111(5):1318–28. doi:10.1080/24694452.2020.1806029
199. Twigt M. Doing Refugee Right(s) with Technologies? Humanitarian Crises and the Multiplication of “Exceptional” Legal States. Refugee Survey Quarterly. 2024;43(1):1-21. doi: 10.1093/rsq/hdad020.
200. Ugwu OP-C, Alum EU, Ugwu JN, Eze VH, Ugwu CN, Ogenyi FC, et al. Harnessing technology for infectious disease response in conflict zones: Challenges, innovations, and policy implications. Medicine. 2024;103(28). doi:10.1097/md.0000000000038834
201. Ungar M, Seymour A. Access Without Borders: A Scoping Review to Identify Solutions to Creating Portable Identity, Education and Health Records for Refugee Children. 2024; doi:10.21203/rs.3.rs-3837529/v1
202. Vahedi L, Qushua N, Seff I, Doering M, Stoll C, Bartels SA, et al. Methodological and Ethical Implications of Using Remote Data Collection Tools to Measure Sexual and Reproductive Health and Gender-Based Violence Outcomes among Women and Girls in Humanitarian and Fragile Settings: A Mixed Methods Systematic Review of Peer-Reviewed Research. Trauma, Violence, & Abuse. 2022;24(4):2498–529. doi:10.1177/15248380221097439
203. Van den Homberg MJ, Gevaert CM, Georgiadou Y. The Changing Face of Accountability in Humanitarianism: Using Artificial Intelligence for Anticipatory Action. Politics and Governance. 2020;8(4):456–67. doi:10.17645/pag.v8i4.3158
204. van Wynsberghe A, Comes T (2020) Drones in humanitarian contexts, robot ethics, and the human–robot interaction. Ethics Inf Technol 22:43–53
205. Vannini S, Gomez R, Lopez D, Mora S, Morrison JC, Tanner J, Youkhana L, Vergara G, del Mar Moreno Tafurt M (2020) Humanitarian organizations’ information practices: Procedures and privacy concerns for serving the undocumented. Electron J Inf Syst Dev Ctries 86:1–8
206. Vannini S, Gomez R, Newell BC (2019) Privacy and Security Guidelines for Humanitarian Work with Undocumented Migrants. ICTD ’19 Proc Tenth Int Conf Inf Commun Technol Dev. https://doi.org/10.1145/3287098.3287120
207. Vannini S, Gomez R, Newell BC (2020) “Mind the five”: Guidelines for data privacy and security in humanitarian work with undocumented migrants and other vulnerable populations. J Assoc Inf Sci Technol 71:927–938
208. Vaswani V, Caenazzo L, Congram D. Corpse identification in mass disasters and other violence: The ethical challenges of a humanitarian approach. Forensic Sciences Research. 2023;9(1). doi:10.1093/fsr/owad048
209. Venakata Swamy M, Agarwal N, Ramaswamy S (2014) C-CBPM: collective context based privacy model. J Ambient Intell Humaniz Comput 5:881–895
210. Vera Espinoza M, Fernández de la Reguera A, Palla I, Bengochea J. The ethics of online data collection: Reflections on key informant interviews researching the impacts of COVID-19 on migrants in Latin America. Migration Letters. 2023;20(2). doi:10.33182/ml.v20i2.2838
211. Voigt S, Schoepfer E, Fourie C, Mager A (2014) Towards semi-automated satellite mapping for humanitarian situational awareness. Proc 4th IEEE Glob Humanit Technol Conf GHTC 2014 412–416
212. Wang B, Lueks W, Sukaitis J, Narbel VG, Troncoso C. Not Yet Another Digital ID: Privacy-Preserving Humanitarian Aid Distribution. 2023 IEEE Symposium on Security and Privacy (SP). 2023;645–63. doi:10.1109/sp46215.2023.10179306
213. Wang N, Christen M, Hunt M, Biller-Andorno N. Supporting value sensitivity in the humanitarian use of drones through an ethics assessment framework. International Review of the Red Cross. 2022;104(919):1397–428. doi:10.1017/s1816383121000989
214. Wang N. “A Success Story that Can Be Sold”? A Case Study of Humanitarian Use of Drones. 2019 IEEE International Symposium on Technology and Society (ISTAS). 2019;1–6. doi:10.1109/istas48451.2019.8938015
215. Watson H, Rodrigues R (2018) Bringing Privacy into the Fold: Considerations for the Use of Social Media in Crisis Management. J Contingencies Cris Manag 26:89–98
216. Wozniak S, Rossberg M, Schaefer G (2013) Towards Trustworthy Mobile Social Networking Services for Disaster Response. 2013 IEEE Int. Conf. Pervasive Comput. Commun. Work.
217. Yang Y, Zhang C, Fan C, Mostafavi A, Hu X. Towards Fairness-Aware Disaster Informatics: an Interdisciplinary Perspective. IEEE Access. 2020;8:201040–54. doi:10.1109/access.2020.3035714
218. Zadhy-Çepoğlu AN. Reflexive reciprocity under an ethics of care: Reflections from the field for Refugee Studies. Migration Studies. 2023;12(3). doi:10.1093/migration/mnad037
